# The importance of blood pressure thresholds versus predicted cardiovascular risk on subsequent rates of cardiovascular disease: a cohort study in English primary care

**DOI:** 10.1016/S2666-7568(21)00281-6

**Published:** 2022-01

**Authors:** Emily Herrett, Helen Strongman, Sarah Gadd, Laurie Tomlinson, Dorothea Nitsch, Krishnan Bhaskaran, Elizabeth Williamson, Tjeerd van Staa, Reecha Sofat, Adam Timmis, Susan Wells, Liam Smeeth, Rod Jackson

**Affiliations:** a Department of Non-Communicable Diseases Epidemiology, London School of Hygiene & Tropical Medicine, London, UK; b Department of Medical Statistics, London School of Hygiene & Tropical Medicine, London, UK; c Health Data Research UK London, London, UK; d Centre for Health Informatics, Division of Informatics, Imaging, and Data Science, School of Health Sciences, University of Manchester, Manchester Academic Health Science Centre, Manchester, UK; e Division of Pharmacoepidemiology and Clinical Pharmacology, Utrecht Institute of Pharmaceutical Sciences, Utrecht, Netherlands; f Institute of Health Informatics, University College London, London, UK; g Queen Mary University London, London, UK; h Section of Epidemiology and Biostatistics, School of Population Health, University of Auckland, Auckland, New Zealand

## Abstract

**Background:**

For five decades, blood pressure lowering treatment has been recommended for patients with hypertension (currently defined as blood pressure of ≥140/90 mm Hg). In the past 20 years, guidelines for treatment began incorporating predicted absolute cardiovascular disease risk (predicted risk) and reducing blood pressure thresholds. The blood pressure threshold at which to start treatment has become a secondary consideration in some countries. We aimed to provide descriptive data to assess the relative importance of blood pressure thresholds versus predicted risk on the subsequent rate of cardiovascular disease to inform treatment decisions.

**Methods:**

In this English population-based cohort study, we used linked data from the Clinical Practice Research Datalink (CPRD) GOLD, Hospital Episode Statistics Admitted Patient Care, and the Office for National Statistics mortality data, and area-based deprivation indices (Townsend scores). Eligible patients were aged 30–79 years on Jan 1, 2011 (cohort entry date) and could be linked to hospital, mortality, and deprivation data. Patients were followed up until death, end of CPRD follow-up, or Nov 31, 2018. We examined three outcomes: cardiovascular disease, markers of potential target organ damage, and incident dementia without a known cause. The rate of each outcome was estimated and stratified by systolic blood pressure and predicted 10-year risk of cardiovascular disease (QRISK2 algorithm).

**Findings:**

Between Jan 1, 2011, and Nov 31, 2018, 1 098 991 patients were included in the cohort and followed up for a median of 4·3 years (IQR 2·6–6·0; total follow-up of 4·6 million person-years). Median age at entry was 52 years (IQR 42–62) and 629 711 (57·3%) patients were female. There were 51 996 cardiovascular disease events and the overall rate of cardiovascular disease was 11·2 per 1000 person-years (95% CI 11·1–11·3). Median QRISK2 10-year predicted risk was 4·6% (IQR 1·4–12·0) and mean systolic blood pressure before cohort entry was 129·1 mm Hg (SD 15·7). Within strata of predicted risk, the effect of increasing systolic blood pressure on outcomes was small. For example, in the group with 10·0–19·9% predicted risk, rates of all cardiovascular disease rose from 20·1 to 23·6 per 1000 person-years between systolic blood pressures less than 110 mm Hg and 180 and higher mm Hg. But among patients with systolic blood pressure 140·0–149·9 mm Hg, rates rose from 6·9 to 52·3 per 1000 person-years between those with less than 10·0% risk and those with 30·0% or higher predicted risk.

**Interpretation:**

For a wide range of blood pressures, the rate of cardiovascular disease and effectiveness of blood pressure drug treatment was mainly determined by predicted risk, with blood pressure thresholds 140/90 mm Hg or 160/100 mm Hg—ubiquitous in most countries—adding little useful information. When medium-term predicted risk is low, there is no urgency to initiate drug treatment, allowing time to attempt non-pharmacological blood pressure reduction.

**Funding:**

National Institute for Health Research.

## Introduction

Blood pressure lowering treatment has been a cornerstone of cardiovascular disease prevention for more than 50 years and for several decades most international guidelines have recommended blood pressure lowering treatment for patients with hypertension (blood pressure ≥140/90 mm Hg).^1^, ^2^, ^3^, ^4^ The distribution of risk factors across populations has changed over time,^5^ and the understanding of predicted absolute cardiovascular disease risk (herein referred to as predicted risk) and effectiveness of blood pressure lowering treatment across different groups have also changed.^6^, ^7^, ^8^, ^9^ In the past two decades, and in light of new evidence, guidelines for blood pressure lowering have changed to target treatment towards patients who might benefit the most.

Two major changes have occurred. First, most guidelines now incorporate predicted risk to guide treatment decisions in patients with mild hypertension (blood pressures between 130/80 mm Hg or 140/90 mm Hg and 159/99 mm Hg).^1^, ^2^, ^3^, ^4^, ^10^ There is some evidence^11^ that an entirely risk-based approach to treatment could be favourable and this approach is taken for most patients with normal blood pressure or mild hypertension in New Zealand.^3^

**Research in context**

**Evidence before this study**

A 2021, individual patient meta-analysis showed that a 5 mm Hg reduction in systolic blood pressure led to a reduction in risk of cardiovascular disease (relative risk 0·91, 95% CI 0·89–0·94) across the blood pressure spectrum. This finding indicates that patients with the highest predicted risk of cardiovascular disease, irrespective of baseline blood pressure, would benefit most from blood pressure lowering treatment.We reviewed global blood pressure treatment guidelines for the primary prevention of cardiovascular disease. We found that absolute predicted cardiovascular risk is now commonly used, in addition to blood pressure, to help guide treatment. However, there is still a focus on hypertension, blood pressure cutoffs, and on targets and control of blood pressure as the key goals to lower cardiovascular disease incidence.Given the results of the meta-analysis, guideline committees and clinicians need a clear understanding of the relative importance of blood pressure and predicted risk on the subsequent rate of cardiovascular disease and target organ damage.

**Added value of this study**

Our study indicates that emphasis on blood pressure targets and control should not detract from focus on an overall reduction in predicted risk. Substantial gains could be made to reduce the burden of cardiovascular disease by treating a large group of patients with high predicted risk.

**Implications of all the available evidence**

This study and the evidence to date indicate that a shift is required to focus away from blood pressure cutoffs and goals, and towards viewing blood pressure in the context of predicted risk for optimal cardiovascular disease prevention.

Second, in the USA, the blood pressure lowering treatment threshold was reduced in 2017, from 140/90 mm Hg to 130/80 mm Hg, on the basis of findings from the SPRINT trial.^10^, ^12^ As the guidelines and supporting data evolve, most countries still focus on the 140/90 mm Hg hypertension cutoff, and on targets and control of blood pressure as the key goals to lower cardiovascular disease incidence. However, viewing hypertension in the context of a patient's predicted risk is crucial. To make good treatment decisions for individual patients and reduce cardiovascular disease burden at the population level, clinicians and patients need to understand how both blood pressure and predicted risk affect the incidence of disease.

This study aims to provide descriptive data to show the relative importance of blood pressure thresholds and predicted risk on the subsequent rate of cardiovascular disease, to guide the use of blood pressure lowering drugs in a contemporary population.

## Methods

### Data sources

In this population-based cohort study, we used linked data from the UK Clinical Practice Research Datalink (CPRD) GOLD, Hospital Episode Statistics (HES) Admitted Patient Care,^13^ and the Office for National Statistics (ONS) mortality data, and area-based deprivation indices (Townsend scores from 1 [least deprived] to 5 [most deprived]).^14^ CPRD GOLD is a well validated UK primary care database containing anonymised patient records including diagnoses, tests, clinical measurements, prescriptions, and specialist referrals.^15^, ^16^ The HES Admitted Patient Care dataset contains diagnoses and procedures from English hospitals^13^ and ONS mortality data contains the date and cause of death.

### Cohort selection and criteria

Eligible patients were aged 30–79 years on Jan 1, 2011 (cohort entry date) and could be linked to hospital, mortality, and deprivation data. This age range was chosen because patients younger than 30 years would rarely meet treatment eligibility criteria and those older than 79 years required careful consideration and were more likely to be treated outside of guideline recommendations. The cohort entry date was chosen to allow sufficient follow-up to accrue outcome events. Patients with follow-up of less than 1 year before cohort entry, an existing diagnosis of cardiovascular disease or target organ damage, fewer than two blood pressure measures before the start of follow-up, or with the most up-to-date blood pressure measure taken more than 5 years before cohort entry were excluded (appendix p 1). Patients with recorded dementia at cohort entry were excluded from analysis estimating the rate of dementia. Patients were followed up until death, end of CPRD follow-up, or Nov 31, 2018 (the last date that linked data from HES were available), whichever occurred first.

Ethical approval was granted by the London School of Hygiene & Tropical Medicine Ethics Committee (reference 22955). Study protocol approval was granted by the Independent Scientific Advisory Committee (protocol 19_113).

### Outcomes

This study examined three outcomes: cardiovascular disease; markers of potential target organ damage; and incident dementia without a known cause. Cardiovascular disease included any record of coronary heart disease (myocardial infarction, angina, revascularisation procedures, and coronary heart disease not otherwise specified), atherosclerotic cerebrovascular disease (including transient ischaemic attack, non-stroke cerebrovascular disease, and stroke [excluding haemorrhagic stroke]), peripheral arterial disease, and heart failure. Markers of potential target organ damage, which encompass damage to the brain, heart, eyes, and kidneys, are thought to be caused by high blood pressure. This outcome included haemorrhagic stroke and chronic kidney disease (there were too few hypertensive retinopathy events in the dataset to analyse and left ventricular hypertrophy is not well recorded in electronic health records). The outcome of incident dementia without a known cause excluded alcohol or drug-induced dementia, Parkinson's disease dementia, Pick's disease, and Huntington's disease. Outcome data were extracted from CPRD, HES and ONS, with the exception of chronic kidney disease which was only extracted from the CPRD database. We used Read and International Classification of Diseases-10 codes to define outcomes. We used coded chronic kidney disease, which in the UK has been incentivised to be recorded as part of the Quality and Outcomes Framework in primary care, rather than estimated glomerular filtration rate (eGFR), because coding implies the general practitioner is aware of the condition and will incorporate it into clinical decision making.^17^

### Exposures and covariates

Systolic and diastolic blood pressures were extracted from CPRD primary care records. For analysis, the mean of the last two blood pressure readings within the past 5 years was categorised into prespecified 10 mm Hg bands (systolic <110·0, 110·0–119·9, 120·0–129·9, 130·0–139·9, 140·0–149·9, 150·0–159·9, 160·0–169·9, 170·0–179·9, ≥180·0 mm Hg; diastolic <70·0, 70·0–79·9, 80·0–89·9, 90·0–99·9, 100·0–109·9, ≥110·0 mm Hg). Blood pressures extracted for this study were from treated and untreated patients. 10-year predicted risk was measured using QRISK2^18^ at cohort entry. Pretreatment blood pressure was not estimated because QRISK2 (2017) incorporates current blood pressure and use of blood pressure lowering drugs; additionally, among those patients treated for hypertension, many do not achieve blood pressure at less than 140/90 mm Hg and further lowering is likely to be effective at reducing cardiovascular disease.^12^ QRISK2 scores were calculated for each patient based on data available in CPRD medical records and linked Townsend scores, using software in the QRISK bundle (version 2.0). QRISK2 scores were categorised into prespecified groups (<10·0%, 10·0–19·9%, 20·0–29·9%, and ≥30·0%) for 10-year predicted risk. The algorithm to calculate QRISK2 was designed to replace missing data with the same values as the online calculator to obtain a score for all patients. Prescription of blood pressure lowering treatment was assessed at cohort entry on the basis of the most recent ongoing prescription. Rheumatoid arthritis was included as part of the QRISK2 algorithm, but other inflammatory diseases associated with cardiovascular disease were not included.

### Statistical analysis

The rate of each outcome (cardiovascular disease, markers of potential target organ damage, and incident dementia without a known cause) was estimated and stratified by systolic blood pressure and predicted risk of cardiovascular disease. In secondary analyses, rates were stratified by blood pressure treatment use at cohort entry (defined by the QRISK2 algorithm), sex, age (≥60 years *vs* <60 years), and diabetes status at cohort entry (as defined by the QRISK2 algorithm). We did not undertake regression analysis or adjust for confounding factors; our analysis was descriptive because the aim was to show differences in rates of outcome for patients in strata to reflect the clinical context in which decisions about treatment are made.

In post-hoc analysis, we calculated rate differences for patients with high systolic blood pressure (≥160 mm Hg) and low predicted risk (<10%) and compared with those with normal systolic blood pressure (<140 mm Hg) and high predicted risk (≥20%).

We additionally conducted a prespecified analysis of diastolic blood pressure in 10 mm Hg bands.

As coded chronic kidney disease underestimates kidney disease prevalence in primary care, we conducted a sensitivity analysis of chronic kidney disease, defined by an eGFR of less than 60 mL/min per 1·73 m^2^ (the most recent measure before cohort entry). The need for repeated measures of blood pressure can introduce survivor bias or restrict to those who visit the general practitioner frequently; thus, in sensitivity analysis we used the most recent measure of blood pressure.

### Role of the funding source

The funder of the study had no role in study design, data collection, data analysis, data interpretation, or writing of the report.

## Results

**Figure 1:**
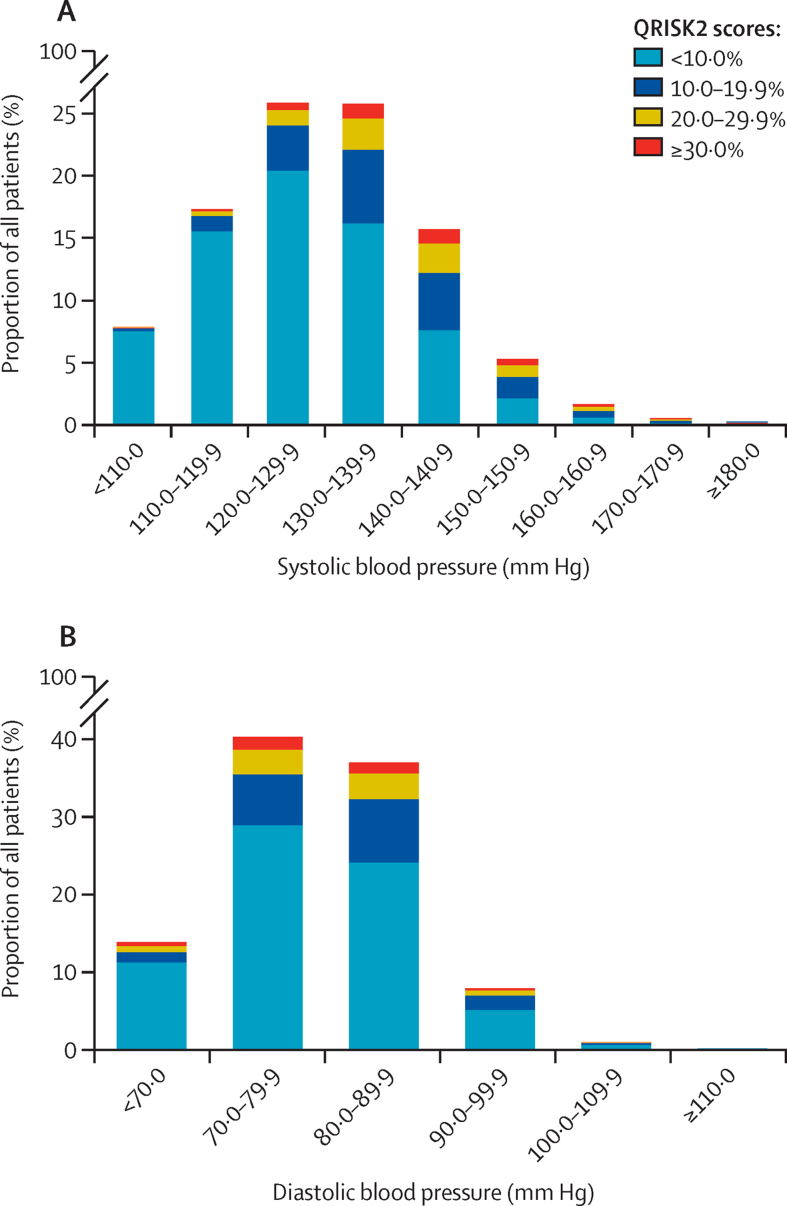
Distribution of systolic blood pressure (A), diastolic blood pressure (B), and predicted 10-year cardiovascular disease risk at cohort entry (n=1·1 million)

Between Jan 1, 2011, and Nov 31, 2018, 1 098 991 patients were included in the cohort and followed up for a median of 4·3 years (IQR 2·6–6·0; total follow-up of 4·6 million person-years). Of 2 671 067 patients assessed for eligibility, 1 572 076 were ineligible because they were younger than 30 years or older than 79 years; did not have a blood pressure record; or had fewer than two blood pressure readings, cardiovascular disease, or target organ damage before cohort entry (appendix p 1). There were 51 996 cardiovascular disease events and the overall rate of cardiovascular disease was 11·2 per 1000 person-years (95% CI 11·1–11·3). Cohort characteristics are described in table 1. Median age at entry was 52 years (IQR 42–62). Median QRISK2 10-year predicted risk was 4·6% (IQR 1·4–12·0) and mean systolic blood pressure before cohort entry was 129·1 mm Hg (SD 15·7). The distribution of systolic and diastolic blood pressure and predicted risk at cohort entry is shown in figure 1.

**Table 1:** Cohort characteristics at cohort entry (Jan 1, 2011)

		**Cohort total (n=1 098 991)**
Follow-up, years		4·3 (2·6–6·0)
Sex		
	Male	469 280 (42·7%)
	Female	629 711 (57·3%)
Age group at cohort entry, years		
	30–39	229 075 (20·8%)
	40–49	289 570 (26·3%)
	50–59	259 279 (23·6%)
	60–69	213 055 (19·4%)
	70–79	108 012 (9·8%)
Ethnicity		
	White or unknown	842 868 (76·7%)
	Indian	13 715 (1·2%)
	Pakistani	5140 (0·5%)
	Bangladeshi	1689 (0·2%)
	Asian other (not specified)	9282 (0·8%)
	Black Caribbean	5488 (0·5%)
	Black African	9202 (0·8%)
	Chinese	2618 (0·2%)
	Other (not specified)	208 989 (19·0%)
QRISK2 score		
	<10·0%	769 101 (70·0%)
	10·0–19·9%	199 493 (18·2%)
	20·0–29·9%	87 865 (8·0%)
	≥30·0%	42 532 (3·9%)
Townsend score		
	1 (least deprived)	283 759 (25·8%)
	2	194 915 (17·7%)
	3	234 464 (21·3%)
	4	211 853 (19·3%)
	5 (most deprived)	174 000 (15·8%)
Family history of cardiovascular disease		89 726 (8·2%)
Diabetes		53 803 (4·9%)
Smoking status		
	Non-smoker	615 768 (56·0%)
	Ex-smoker	260 126 (23·7%)
	Light smoker	51 922 (4·7%)
	Moderate smoker	118 008 (10·7%)
	Heavy smoker	45 297 (4·1%)
	Unknown	7870 (0·7%)
Body-mass index		26·6 (23·8–30·2)
	Unknown	128 108 (11·7%)
Total; HDL cholesterol ratio		3·9 (1·2)
	Unknown	621 290 (56·5%)
Systolic blood pressure, mm Hg		
	<110·0	86 327 (7·9%)
	110·0–119·9	189 760 (17·3%)
	120·0–139·9	566 423 (51·5%)
	140·0–159·9	230 231 (20·9%)
	160·0–179·9	23 617 (2·1%)
	≥180·0	2633 (0·2%)
Mean systolic blood pressure, mm Hg		129·1 (15·7)
Mean diastolic blood pressure, mm Hg		78·0 (9·5)
Use blood pressure lowering medication		209 896 (19·1%)
Rheumatoid arthritis		9636 (0·9%)
Atrial fibrillation		10 421 (0·9%)

Data are median (IQR), n (%), or mean (SD). Variables include components of QRISK2,^19^ which handles missing data by imputing average values.

**Figure 2:**
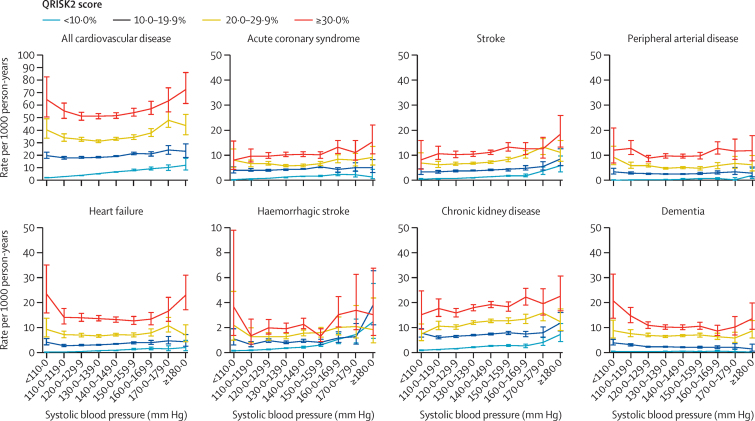
Rate of cardiovascular disease, acute coronary syndrome, stroke, peripheral arterial disease, heart failure, haemorrhagic stroke, chronic kidney disease, and dementia stratified by systolic blood pressure and predicted 10-year cardiovascular disease risk at cohort entry Bars show 95% CIs. All cardiovascular disease includes all coronary heart disease, cerebrovascular disease, peripheral arterial disease, and heart failure.

The rate of outcomes during follow-up, across the blood pressure spectrum and stratified by predicted risk is shown in figure 2. Patterns are similar across all cardiovascular disease and target organ damage outcomes. Crude associations between blood pressure and outcomes are shown in the appendix (p 2) and show increasing rates with increasing blood pressure. However, figure 2 shows that within strata of predicted risk, the effect of increasing systolic blood pressure on outcomes was small. For example, in the group with 10·0–19·9% predicted risk, rates of all cardiovascular disease rose from 20·1 to 23·6 per 1000 person-years between systolic blood pressures less than 110 mm Hg and 180 and higher mm Hg. But among patients with systolic blood pressure 140·0–149·9 mm Hg, rates of all cardiovascular disease rose from 6·9 to 52·3 per 1000 person-years between less than 10·0% and 30·0% and higher predicted risk.

With the exception of haemorrhagic stroke, patients with low predicted risk (<10%) and high systolic blood pressure (≥160 mm Hg) have strikingly lower rates of outcomes than patients with lower blood pressure (<140 mm Hg) and high predicted risk (≥20%; table 2). For haemorrhagic stroke there were few events (n=2677), and, although the rate increased at systolic blood pressures of 180 mm Hg and higher in the lower predicted risk groups (<10·0% and 10·0–19·9%), only 31 events (1·4% of all haemorrhagic stroke) occurred in this stratum.

**Table 2:** Rates of outcomes for patients with QRISK2^19^ 10-year predicted risk and the rate difference

	**Rate of outcome per 1000 person-years (95% CI)**		**Rate difference**
	Low QRISK2 (<10%) and systolic blood pressure (≥160 mm Hg; n=8064)	High QRISK2 (≥20%) and systolic blood pressure (<140 mm Hg; n=67 202)	
All cardiovascular disease	9·9 (8·9 to 11·0)	38·2 (37·5 to 39)	28·3 (27·0 to 29·6)
Acute coronary syndrome	2·6 (2·1 to 3·2)	7·5 (7·2 to 7·9)	5·0 (4·3 to 5·6)
Stroke	2·6 (2·1 to 3·2)	7·9 (7·6 to 8·3)	5·3 (4·7 to 6·0)
Peripheral arterial disease	0·8 (0·6 to 1·2)	6·7 (6·4 to 7·0)	5·9 (5·5 to 6·3)
Heart failure	1·7 (1·4 to 2·2)	9·0 (8·6 to 9·3)	7·2 (6·7 to 7·8)
Haemorrhagic stroke	1·2 (0·9 to 1·7)	1·5 (1·3 to 1·6)	0·3 (−0·1 to 0·6)
Chronic kidney disease	3·2 (2·6 to 3·8)	13·0 (12·6 to 13·5)	9·9 (9·1 to 10·6)
Dementia	0·3 (0·2 to 0·6)	7·8 (7·5 to 8·1)	7·5 (7·1 to 7·9)

For the composite cardiovascular disease outcome (heart failure and dementia), J-shaped curves were observed which were accentuated in the highest predicted risk group (≥30·0%; figure 2).

The numbers and proportion of events occurring according to blood pressure and predicted 10-year cardiovascular risk are described in the appendix (pp 3–6). Most outcomes occurred among patients with systolic blood pressures 120·0–159·9 mm Hg and diastolic blood pressures 70·0–89·9 mm Hg; overt target organ damage is rare in this blood pressure range.

209 896 (19·1%) of 1 098 991 patients were using blood pressure lowering medication at cohort entry (132 260 additional patients received at least one prescription during follow-up). There were no substantive differences in rates by predicted risk or systolic blood pressure among patients given blood pressure lowering drugs at cohort entry and those not given such drugs, except for chronic kidney disease, which showed higher rates in patients prescribed blood pressure lowering drugs (appendix p 7).

Women had lower rates of cardiovascular disease overall, but higher rates of dementia and chronic kidney disease, than men in the same stratum of predicted risk and systolic blood pressure (appendix p 8). When stratified by age (<60 *vs* ≥60), the older age group retained the J-shaped curves observed in the main analysis (appendix p 9). For patients younger than 60 years, there was less power (ie, fewer events) and so no clear associations were observed. Importantly, there were large increases in the rates of all cardiovascular disease for patients younger than 60 years at high predicted risk (≥30·0%) and systolic blood pressure of more than 160·0 mm Hg, which were not observed in the same age group in lower predicted risk groups (appendix p 9).

53 803 (4·9%) patients with diabetes at cohort entry had similar rates of outcomes to those without diabetes (appendix p 10). At most systolic blood pressures, predicted risk remained important, but there were few events among patients with diabetes at systolic blood pressures higher than 160 mm Hg.

In the sensitivity analysis, the rate of chronic kidney disease per 1000 person-years measured by eGFR (<60 mL/min per 1·73 m^2^) was substantially higher than the rate measured by coded chronic kidney disease in the main analysis. However, the pattern was similar between the coded and eGFR methods across varying systolic blood pressures and predicted cardiovascular disease risk (appendix p 11). Results using the patient's most recent blood pressure instead of the mean of the last two readings showed similar results to the main analysis, but with a shallower gradient between blood pressure and cardiovascular disease risk (in particular heart failure) at higher blood pressures (appendix p 12). Results for diastolic blood pressure are shown in the appendix (p 13). Within strata of predicted risk, the effect of diastolic blood pressure on outcomes was small compared with the impact of predicted risk, However, there were more pronounced increases in rates of outcomes among those with diastolic blood pressures of 110 mm Hg or higher.

## Discussion

Over approximately 4-year follow-up, our analyses showed that once cardiovascular disease risk is accounted for, blood pressure thresholds add little useful information. Our results were similar across systolic and diastolic blood pressures; among patients prescribed or not prescribed blood pressure lowering drugs; and by sex, age, and diabetes status.

We show that predicted risk, which incorporates blood pressure and numerous other risk factors to predict the 10-year risk of coronary heart disease and stroke,^18^ is a better predictor of outcomes than blood pressure alone. The results are unsurprising, given the worldwide adoption of risk calculators in determining use of statin treatment and blood pressure lowering treatment for those with mild hypertension.^2^, ^10^, ^20^ Risk calculators have been available since the 1990s and are widely used in clinical practice.^21^

Similar results were shown nearly 30 years ago in the MRFIT study,^22^ in which the combined effects of smoking, high cholesterol, and high blood pressure led to high rates of coronary heart disease. Our findings also broadly support data from the Prospective Studies Collaboration (Lewington and colleagues)^23^ by showing superiority of cardiovascular disease risk prediction over blood pressure alone, although our study showed J-shaped associations. The study by Lewington and colleagues^23^ showed substantial increases in the rates of stroke and ischaemic heart disease according to age (and smaller increases in these outcomes with increasing blood pressure). Jackson and colleagues^24^ suggested that the clinical terms hypertension and hypercholesterolaemia were limited when used alone, given the importance of predicted risk. In 2018–19, Karmali and colleagues^11^ and a study from our group^19^ showed that strategies for blood pressure lowering were effective on the basis of risk rather than blood pressure alone. There is also evidence that these strategies are more cost-effective.^25^, ^26^ Our study adds an illustration of the differences in rates of cardiovascular disease outcomes—in a contemporary population from routine care in England—and across a broad spectrum of blood pressures and levels of predicted risk. In particular, figure 2 offers an opportunity for clinicians and patients to visualise the importance of predicted risk in the context of blood pressure over 4 years of follow-up.

Although our study shows the importance of predicted risk in understanding future cardiovascular disease and target organ damage, the effectiveness and safety of blood pressure lowering treatment at lower blood pressures remains highly debated. Controversial and conflicting results have arisen from meta-analyses with different methodologies,^7^, ^8^ and from randomised controlled trials.^12^, ^27^ However, an individual patient meta-analysis^9^ has recently shown that blood pressure lowering is effective at less than 140/90 mm Hg, and concluded that there is a fixed relative reduction in cardiovascular events regardless of blood pressure. This finding indicates that further changes in guidelines towards a risk-based approach might be imminent.

Although rare at the population level, patients with extremely high or low systolic blood pressure require special consideration, as those with systolic blood pressures higher than 180 mm Hg seemed to have higher rates of outcomes (cardiovascular disease, target organ damage, and dementia) at all levels of predicted risk in our study, indicating a need for careful management. This finding is particularly important for young patients (<40 years), whose long-term risk of target organ damage might be high if untreated. At systolic blood pressures lower than 120 mm Hg, the increases in rates of all cardiovascular disease, heart failure, and peripheral arterial disease shown in our study are similar to other observational studies, in which the increases can be explained by confounding (high morbidity from cardiovascular and non-cardiovascular causes)^28^ or reverse causality (ie, blood pressures might decline before death).^29^ Although we excluded patients with clinical evidence of cardiovascular disease and target organ damage at cohort entry, we noted the J-shaped curve only in patients aged 60 years and older, in whom blood pressures might have declined as a result of marked vascular damage or left ventricular dysfunction following chronic uncontrolled hypertension or non-cardiovascular morbidities contributing to the risk. Therefore, low blood pressures and a high predicted risk should be investigated to avoid missed opportunities for care.

Although incidence of coded chronic kidney disease was high (even higher incidence with eGFR), there is genetic evidence suggesting that high blood pressure is an early manifestation of underlying kidney damage.^30^ We were unable to look at albuminuria as an early manifestation of kidney disease because of a small number of patients in this cohort tested for albuminuria in primary care. QRISK2 does not include albuminuria; therefore, we might have underestimated the effect of blood pressure-related kidney damage on clinical outcomes.

Our study included 4 million patient-years of follow-up and patients with a wide range of mean systolic blood pressures and predicted risk. The CPRD is broadly representative of the UK population^15^ and we used linkage to hospital and mortality data to improve outcome ascertainment. A median follow-up of 4·3 years makes the presented results useful for decision making in the short and medium term. We excluded patients with diagnosed cardiovascular disease and target organ damage to focus the analysis on incident events. Importantly, our study included patients who present to the general practitioner and have their blood pressure measured, and for whom treatment decisions need to be made.

Restricting the cohort to patients with two or more blood pressure readings resulted in bias towards the inclusion of women, patients who consult their general practitioner more frequently, those who are likely to have higher blood pressures, are older, and have more comorbidities.^31^ Therefore, outcome rates might be higher than in the general population.

There are some limitations of primary care data that are relevant to this study. For example, general practitioners might test kidney function more often in those with higher blood pressure; however, this detection bias would lead to more people with kidney damage being detected in the high blood pressure groups and would not fit the pattern dependent on predicted risk groups. Additionally, for diagnoses such as atrial fibrillation (which requires a confirmatory electrocardiogram), the code list used in this study might not be sensitive enough to capture all cases. However, QRISK2 code lists are not published and, therefore, we cannot confirm the accuracy of replication.

The shallow gradients seen with increasing blood pressure in predicted risk groups are for 4 years of follow-up. In the long term and if left untreated, subclinical vessel damage due to high blood pressure could lead to poor outcomes, particularly stroke and vascular dementia; we also acknowledge that high blood pressure might be easier to treat and control in the early stages and leaving patients untreated for long periods might not be the optimal approach.

QRISK2 is the best available validated tool in the UK primary care to predict cardiovascular risk (QRISK3 has not yet been adopted into clinical practice). No risk tool will perfectly predict whether patients will have an outcome, particularly across all cardiovascular disease.^32^ Other risk scoring tools, such as SCORE or the Framingham Risk Score, are used in other settings, such as Europe and the USA, and incorporate fewer risk factors than QRISK2 (eg, no inclusion of ethnicity, area-based deprivation indices, and different levels of smoking). In our study, a proportion of cholesterol (621 290 [56·5%]), body-mass index (128 108 [11·7%]), and smoking (7870 [0·72%]) values were missing; the QRISK2 algorithm imputes average values for these patients, which will introduce error to the estimates of predicted risk. Additional predictors could be valuable and, for some patients, QRISK2 might predict risk less accurately or other factors should be considered (eg, the risk of adverse effects of treatment).^33^

Although our study mostly focuses on the White English population using the QRISK2 algorithm, because of the large sample size we were able to include a substantial number of people from other ethnic groups, albeit in smaller proportions. The principle of using risk prediction to further guide blood pressure treatment is applicable to any country that incorporates cardiovascular risk estimation into routine care. Other countries will choose the risk prediction tool which is most valid in their context.

The outcomes chosen for this study were cardiovascular disease and target organ damage. We were interested in vascular dementia as an adverse effect of high blood pressure. Unfortunately, in most primary care records, the cause of dementia is unknown and, therefore, we broadened our definition of dementia to include diagnoses with no known cause.

Finally, irrespective of the blood pressure cutoff guidelines for initiating treatment, we need to be mindful that blood pressure targets might be different. Our study did not investigate treatment targets and included patients who were prescribed and not prescribed blood pressure lowering drugs at cohort entry, because for most patients the treatment targets are not reached and evidence suggests lowering blood pressure even further seems not to be harmful.^12^

Our results show that within strata of predicted risk, the treatment cutoffs of 140/90 mm Hg and 160/100 mm Hg—ubiquitous in most countries—are not supported by the data in terms of an increase in rates of cardiovascular disease, target organ damage, and incident dementia compared with lower blood pressures.

Patients and clinicians often focus on blood pressure measurements alone, and the data in this study show the importance of multiple risk factors driving cardiovascular risk. For individual patients making decisions about treatment, the high relative risk associated with high blood pressure should be explained in the context of their predicted absolute risk. Importantly, when medium-term predicted risk is low, there seems to be no urgency to initiate drug treatment (on the basis of low incidence rates of cardiovascular disease among this group) and the clinician might want to focus on advising patients to use non-pharmacological approaches to reduce blood pressure.

At the population level, emphasis on blood pressure targets and control should not detract from focus on overall reduction of predicted risk. Most gains can be made by treating patients at highest predicted risk rather than those with high blood pressure. For blood pressures of 140/90 mm Hg and higher (including those 160/100 mm Hg and higher), although there is robust evidence of treatment benefit, the absolute gain might be small if the predicted risk is low. For blood pressures lower than 140/90 mm Hg, (ie, lower than the current treatment threshold) if there is a real treatment benefit, then substantial gains could be made to reduce the burden of cardiovascular disease by treating a large group of patients with high predicted risk.

For a wide range of blood pressures, medium-term drug treatment decisions could be effectively made on the basis of predicted absolute cardiovascular disease risk because blood pressure thresholds would add little useful information. Although blood pressure is an important part of risk prediction, a shift in the focus away from blood pressure cutoffs and targets and towards viewing blood pressure in the context of predicted risk could lead to a more effective approach for cardiovascular disease prevention.

## Data sharing

Data were obtained from the CPRD, a research service that provides primary care and linked data for public health research. CPRD data governance and our licence to use CPRD data does not allow us to distribute or provide access to patient data to other parties. Researchers can apply for data access at https://www.cprd.com/ and need approval of the study protocol by the Independent Scientific Advisory Committee for MHRA database research.

## Declaration of interests

DN was on the steering group for two GlaxoSmithKline funded studies investigating aspects of kidney function in children and adults in sub-Saharan Africa; holds a grant from the UK Health Foundation on improving acute cardiac care for kidney patients; and is the Renal Association's director of informatics research. LT is a member of three Medicines and Healthcare products Regulatory Agency's expert advisory groups and an unpaid member of two non-industry funded trial advisory committees. EW received personal payment for providing training on statistical methods, outside of the submitted work. All other authors declare no competing interests.
