# Supplementary material for: The importance of blood pressure thresholds versus predicted cardiovascular risk on subsequent rates of cardiovascular disease: a cohort study in English primary care
Source: Lancet Healthy Longev. 2022 Jan;3(1):e22–30. doi: 10.1016/S2666-7568(21)00281-6 (PMC8732286; doi:10.1016/S2666-7568(21)00281-6)
Supplement: Supplementary appendix [file mmc1.pdf]

THE LANCET
Healthy Longevity

Supplementary appendix

This appendix formed part of the original submission and has been peer reviewed. We post it as supplied by the authors.

Supplement to: Herrett E, Strongman H, Gadd S, et al. The importance of blood pressure thresholds versus predicted cardiovascular risk on subsequent rates of cardiovascular disease: a cohort study in English primary. *Lancet Healthy Longev* 2022; **3:** e22–30

Appendices

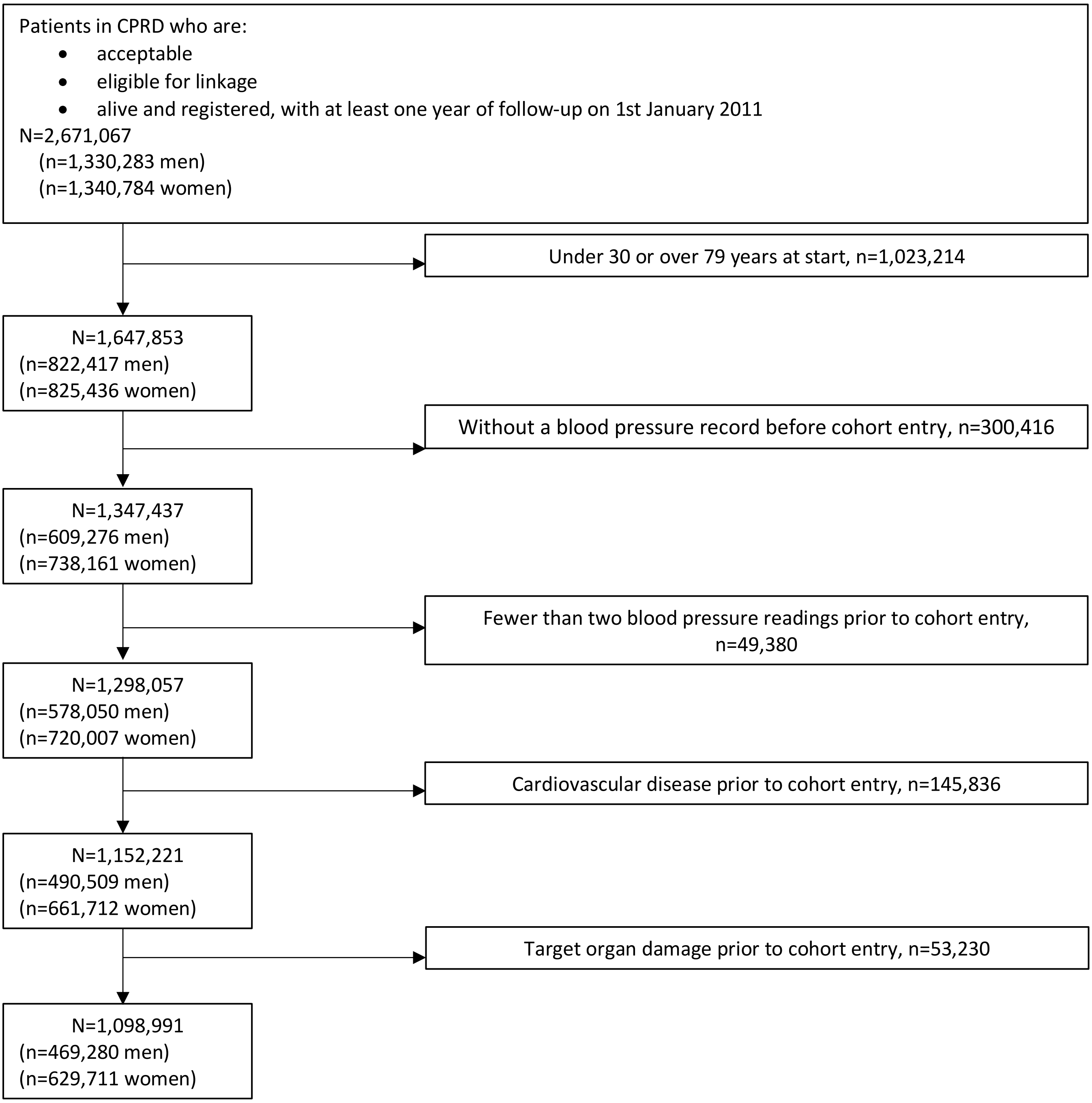

Appendix Figure 1. Flow chart for cohort creation.

A. Systolic blood pressure

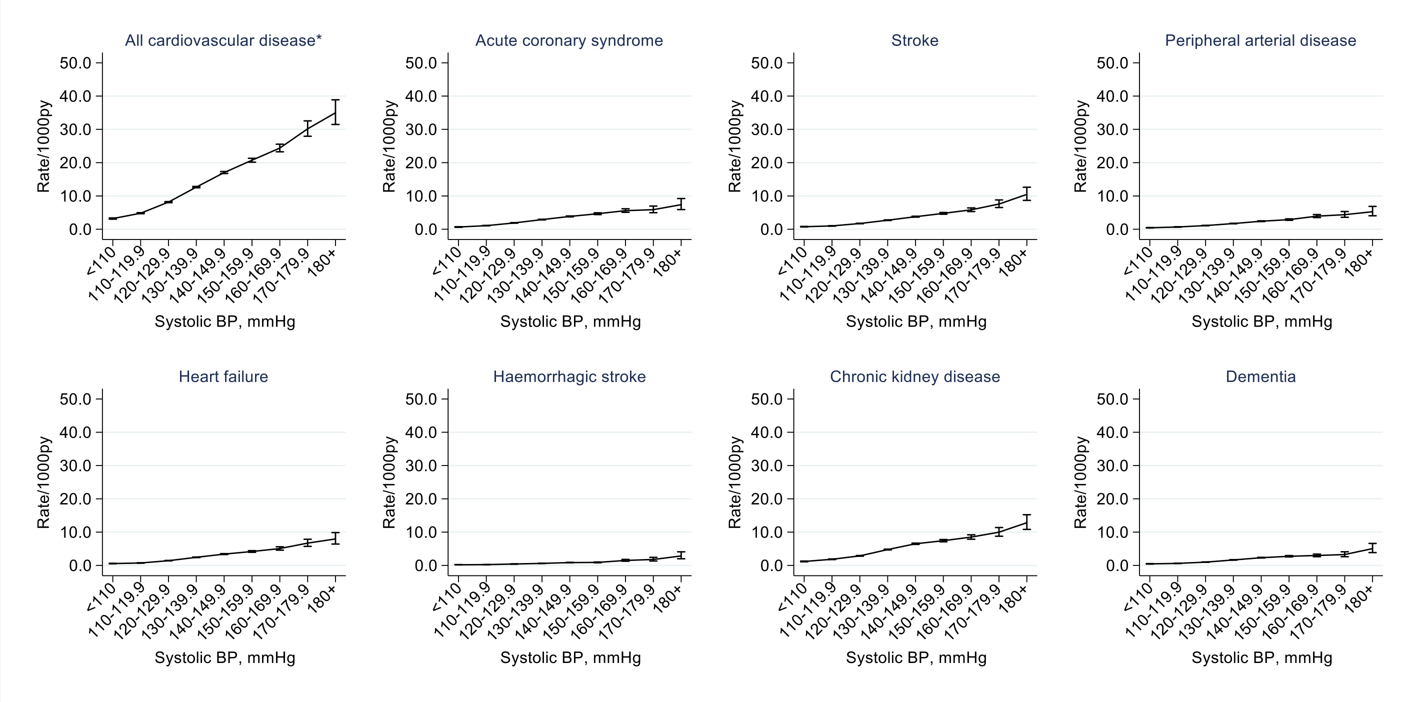

B. Diastolic blood pressure

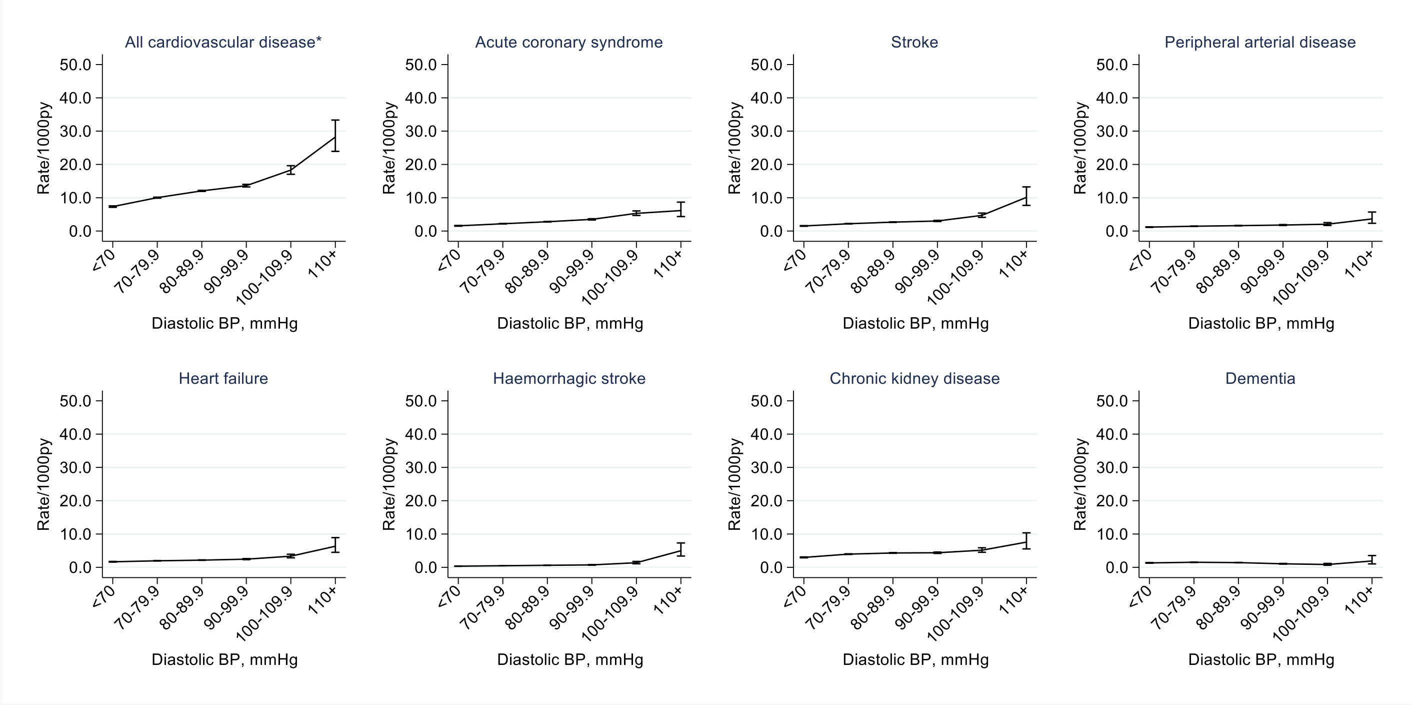

Appendix Figure 2. Crude rates of outcomes by (A) systolic and (B) diastolic blood pressure.
BP: blood pressure
***** All cardiovascular disease includes all coronary heart disease, cerebrovascular disease, peripheral arterial disease and heart failure.

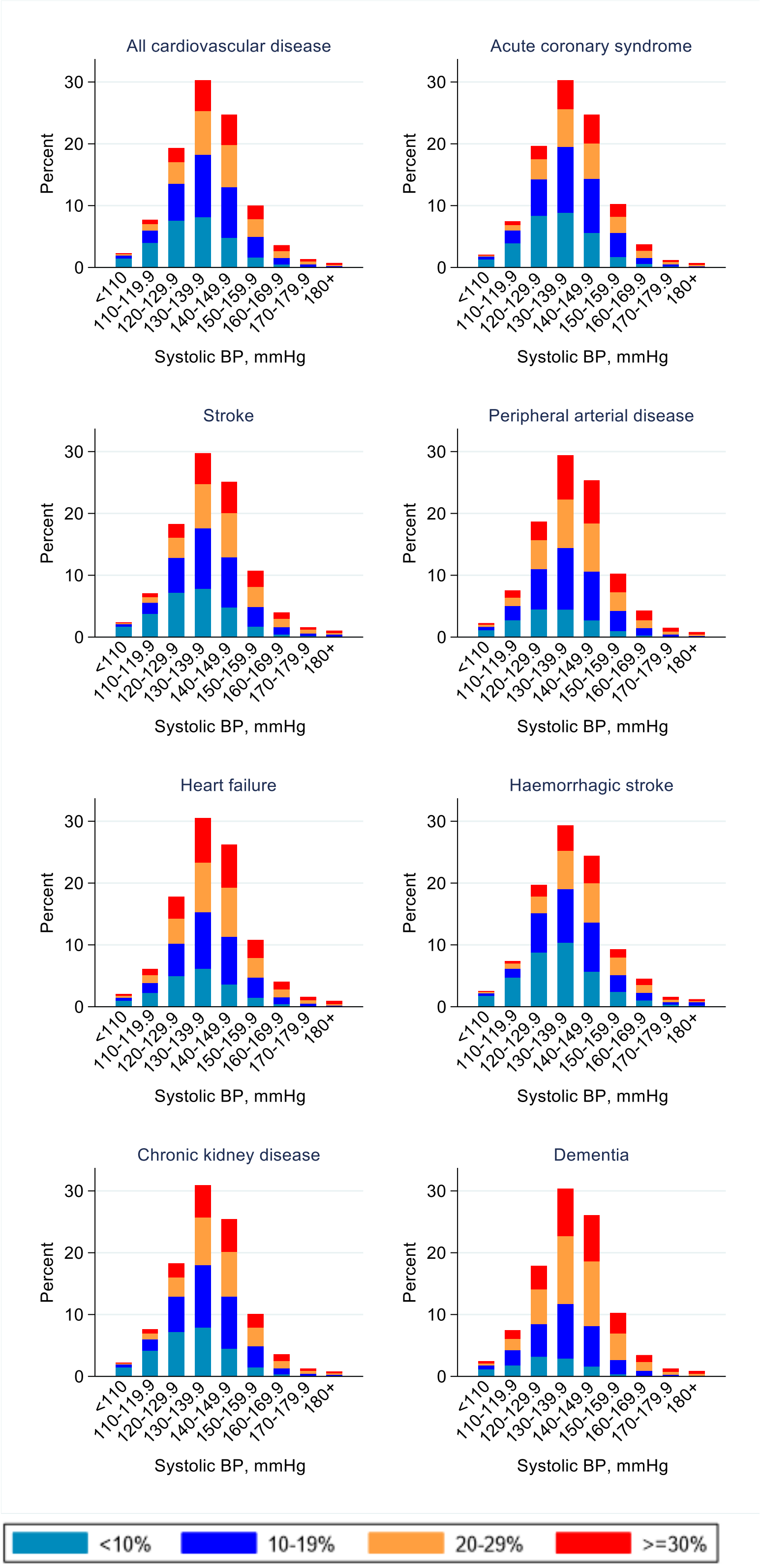

Appendix Figure 3. Proportion of events occurring in patients according to their systolic blood pressure and predicted ten year cardiovascular risk score at cohort entry.

BP: blood pressure

All cardiovascular disease includes all coronary heart disease, cerebrovascular disease, peripheral arterial disease and heart failure.

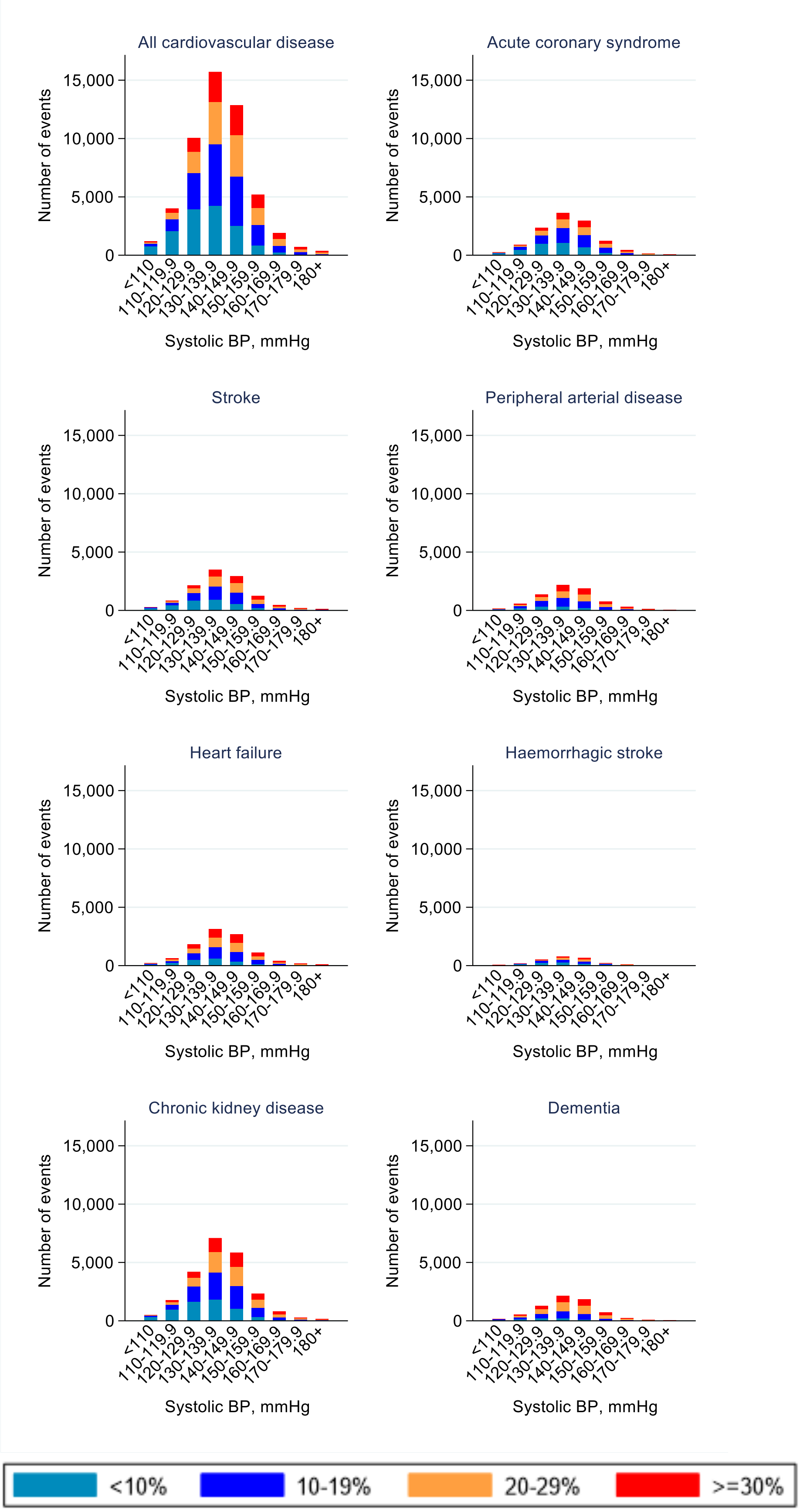

Appendix Figure 4. Number of events occurring in patients according to their systolic blood pressure and predicted ten year cardiovascular risk score at cohort entry.

BP: blood pressure

All cardiovascular disease includes all coronary heart disease, cerebrovascular disease, peripheral arterial disease and heart failure.

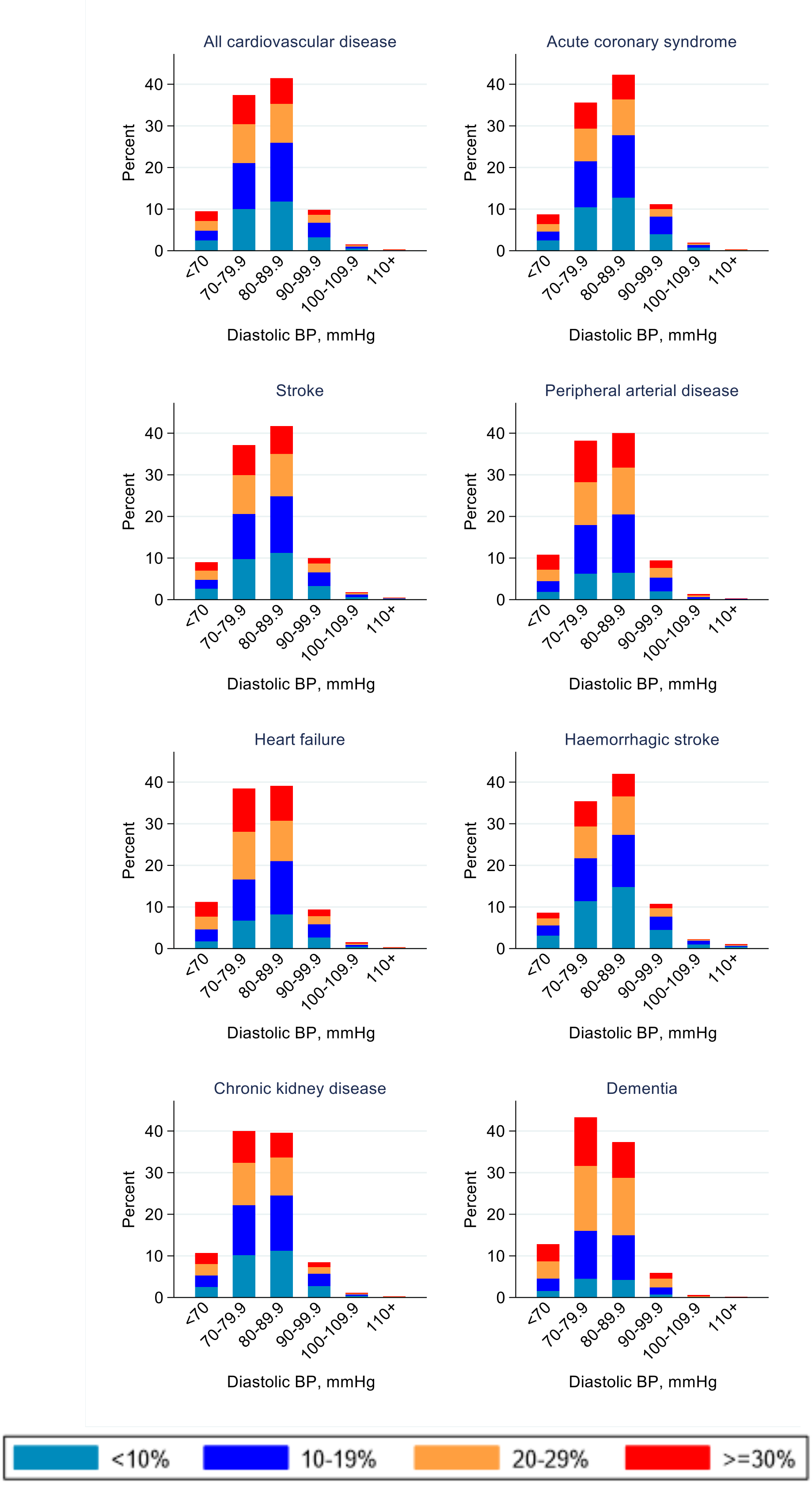

Appendix Figure 5. Proportion of events occurring in patients according to their diastolic blood pressure and predicted ten year cardiovascular risk score at cohort entry.

BP: blood pressure

All cardiovascular disease includes all coronary heart disease, cerebrovascular disease, peripheral arterial disease and heart failure.

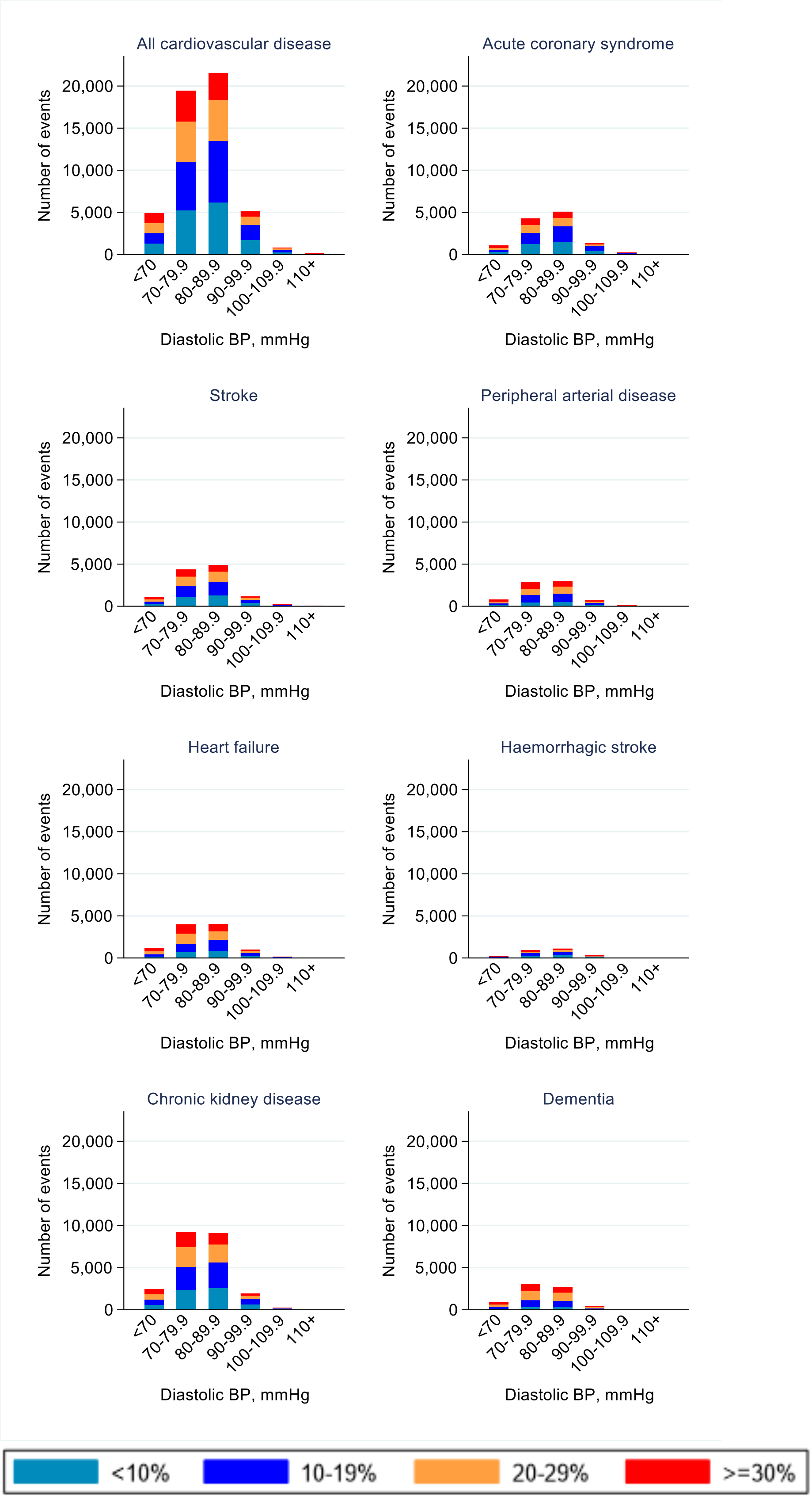

Appendix Figure 6. Number of events occurring in patients according to their diastolic blood pressure and predicted ten year cardiovascular risk score at cohort entry.

BP: blood pressure

All cardiovascular disease includes all coronary heart disease, cerebrovascular disease, peripheral arterial disease and heart failure.

A Treated

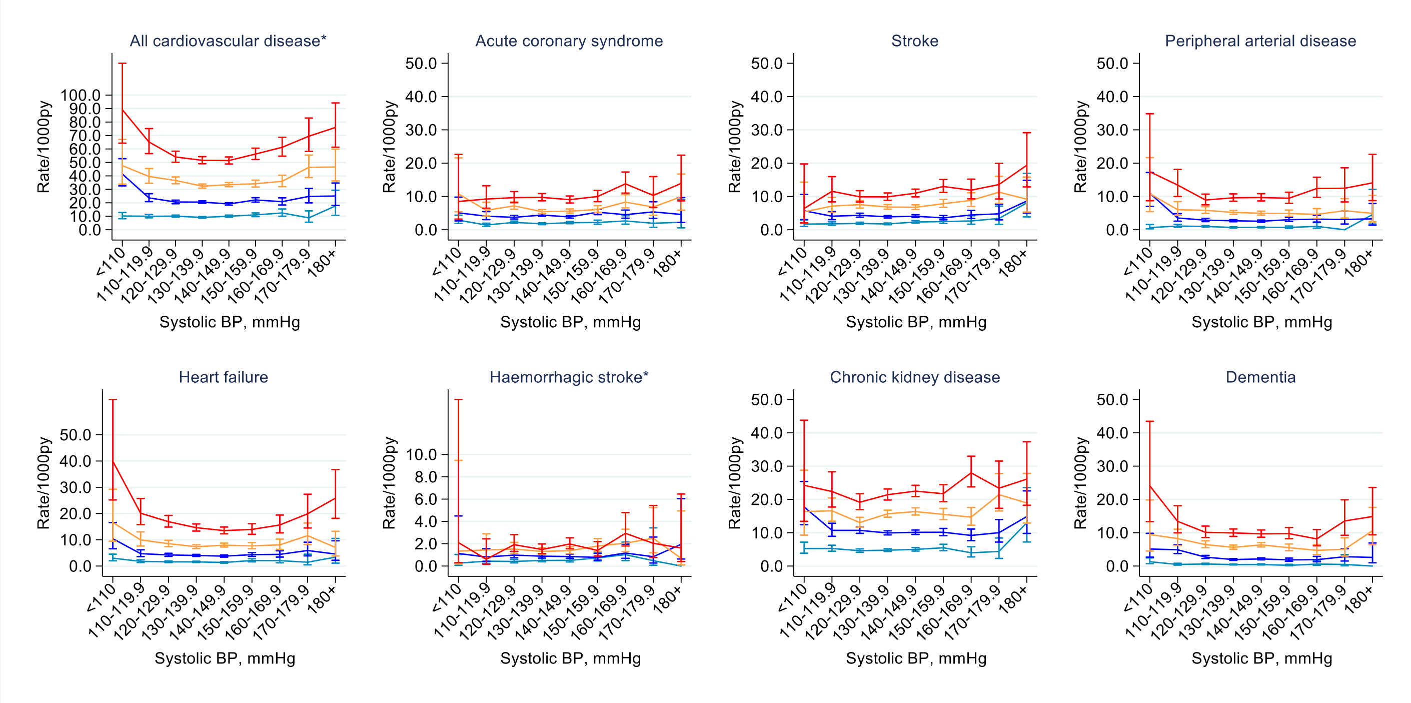

B Untreated

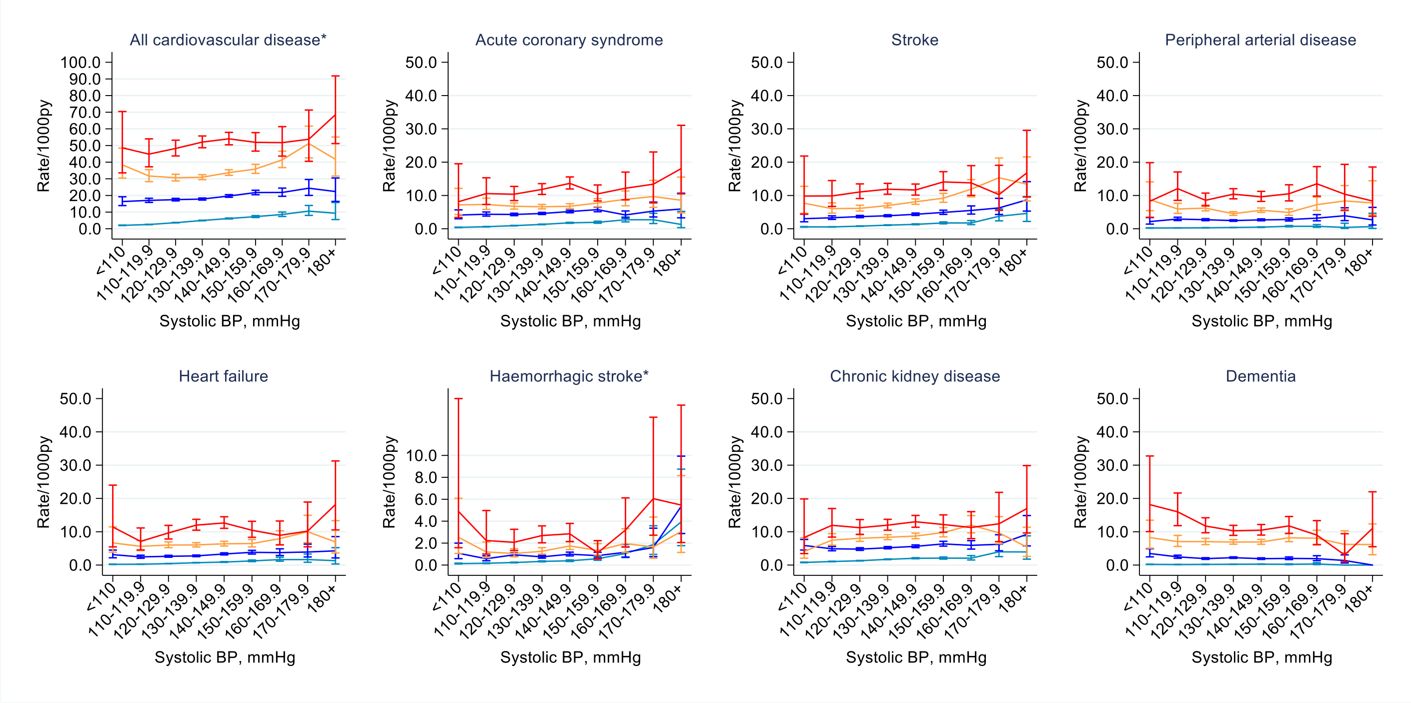

<10% 10-19.9% 20-29.9% >=30% predicted risk

Appendix Figure 7. Among patients who were (A) <u>treated</u> with blood pressure lowering at cohort entry and (B) <u>not treated</u> with blood pressure lowering at cohort entry, the rate (with 95% confidence interval) of cardiovascular disease, acute coronary syndrome, stroke, peripheral arterial disease, heart failure, haemorrhagic stroke, chronic kidney disease and dementia stratified by systolic blood pressure and predicted ten year cardiovascular disease risk (QRISK2) at cohort entry.

BP: blood pressure

*Note different scales on axes for all cardiovascular disease and haemorrhagic stroke. All cardiovascular disease includes all coronary heart disease, cerebrovascular disease, peripheral arterial disease and heart failure.

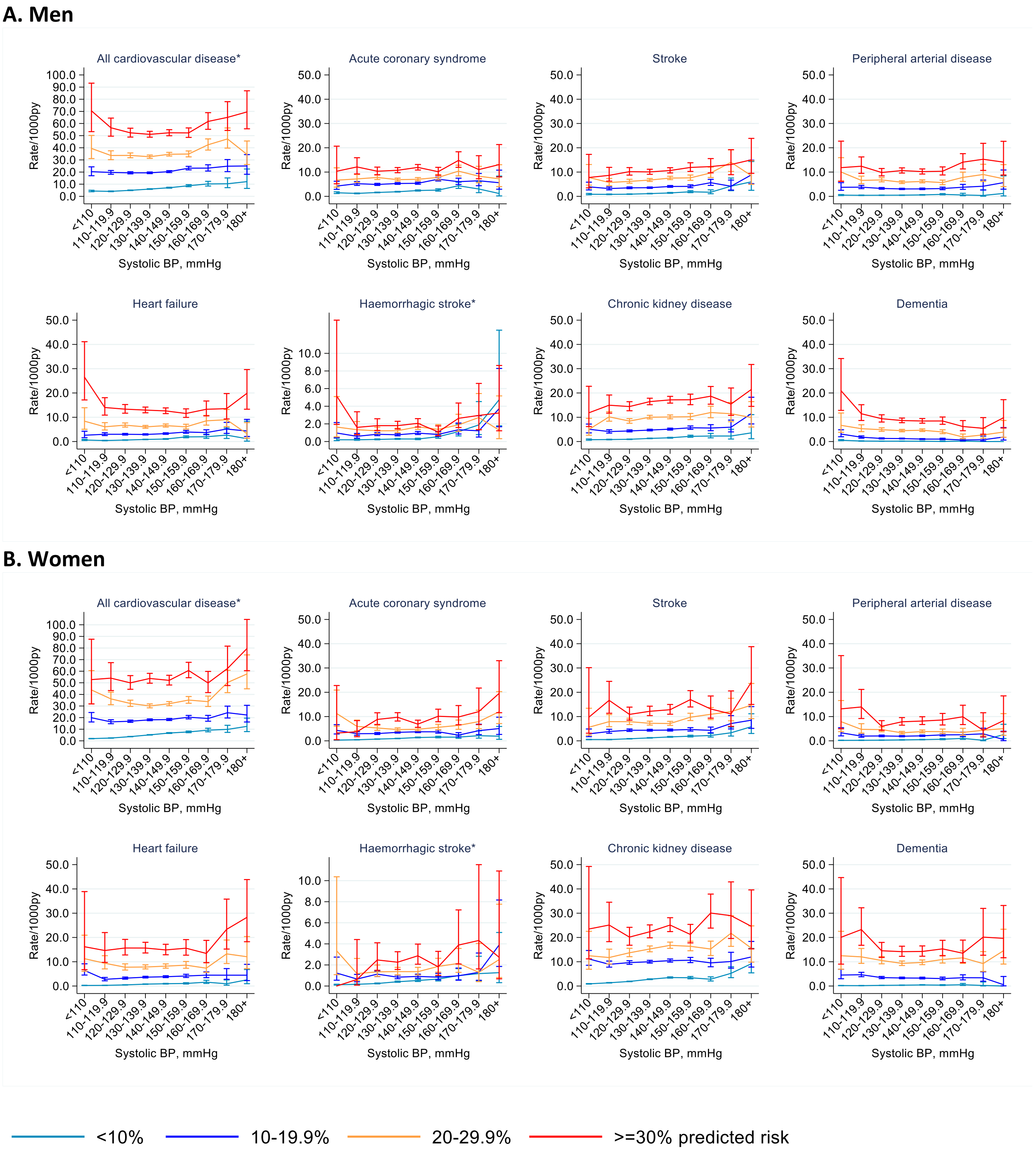

Appendix Figure 8. Among (A) <u>men</u> and (B) <u>women</u>, the rate (with 95% confidence interval) of cardiovascular disease, acute coronary syndrome, stroke, peripheral arterial disease, heart failure, haemorrhagic stroke, chronic kidney disease and dementia stratified by systolic blood pressure and predicted ten year cardiovascular disease risk (QRISK2) at cohort entry.

BP: blood pressure

*Note different scales on axes for all cardiovascular disease and haemorrhagic stroke. All cardiovascular disease includes all coronary heart disease, cerebrovascular disease, peripheral arterial disease and heart failure.

A. Age under 60

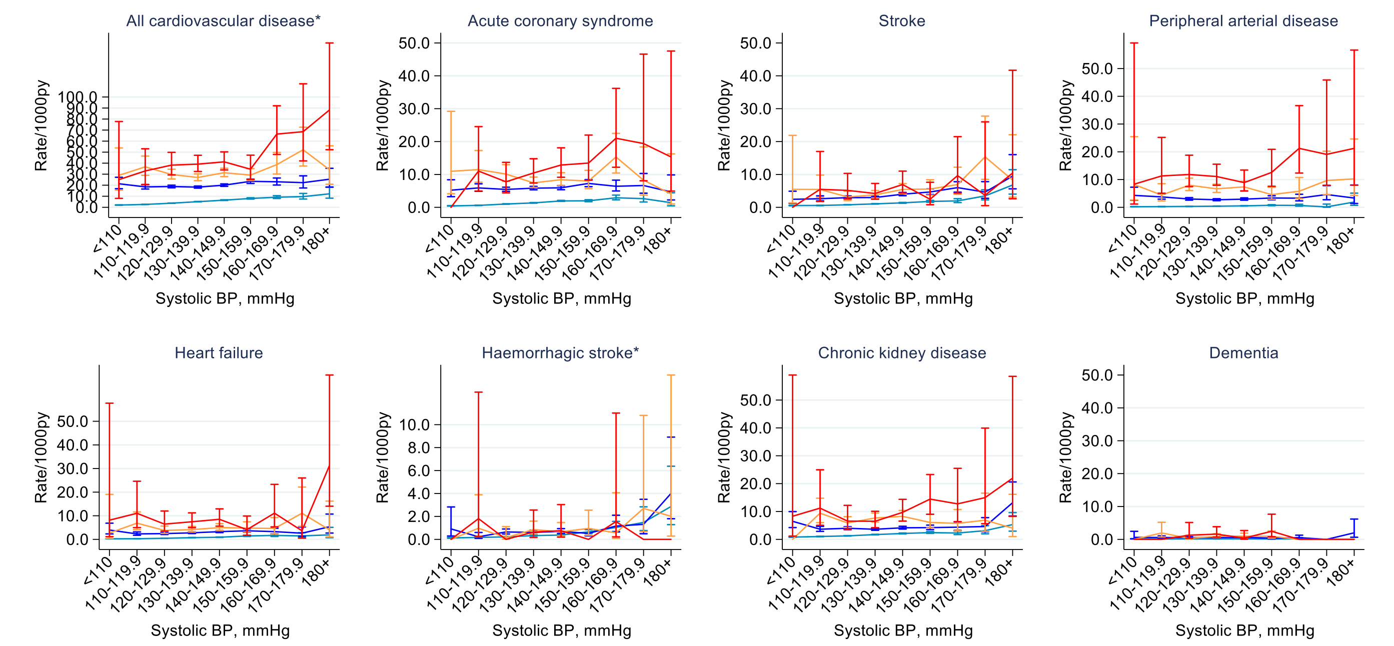

B. Age 60 and over

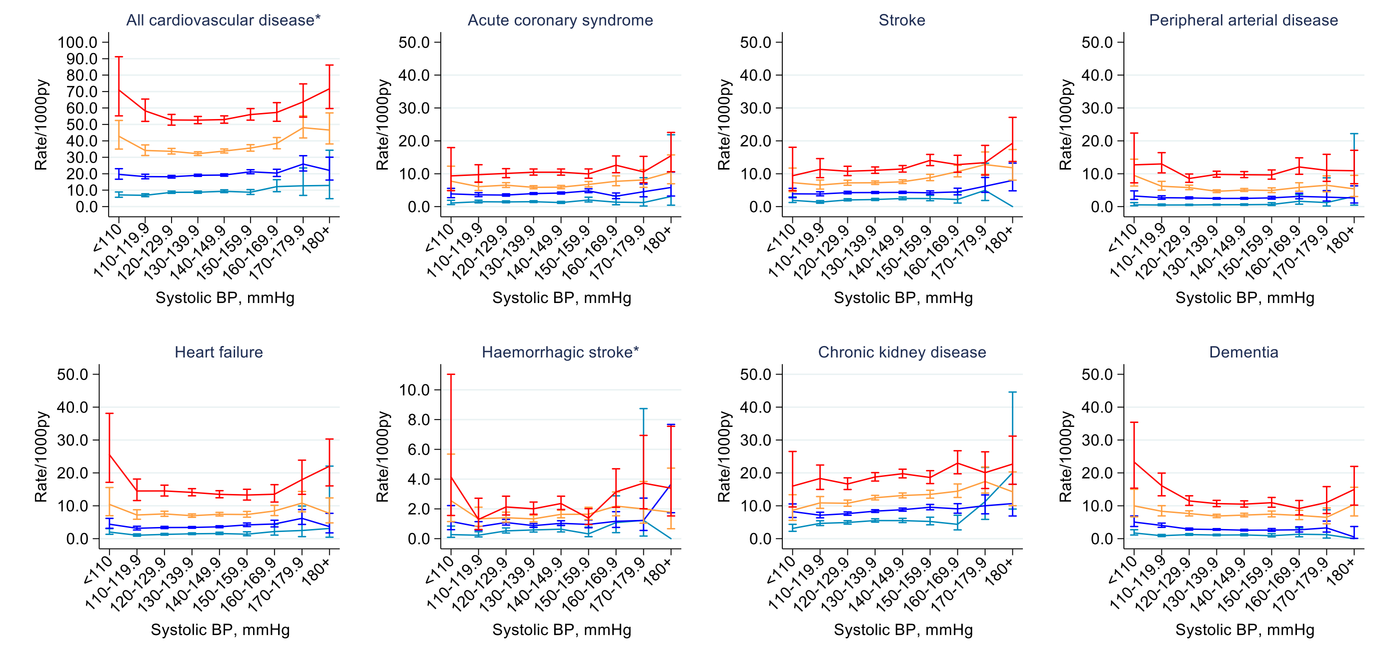

<10% 10-19.9% 20-29.9% >=30% predicted risk

Appendix Figure 9. Among (A) aged under 60 and (B) aged 60 or over, the rate (with 95% confidence interval) of cardiovascular disease, acute coronary syndrome, stroke, peripheral arterial disease, heart failure, haemorrhagic stroke, chronic kidney disease and dementia stratified by systolic blood pressure and predicted ten year cardiovascular disease risk (QRISK2) at cohort entry.

BP: blood pressure

*Note different scales on axes for all cardiovascular disease and haemorrhagic stroke. All cardiovascular disease includes all coronary heart disease, cerebrovascular disease, peripheral arterial disease and heart failure.

A. Diabetes at cohort entry

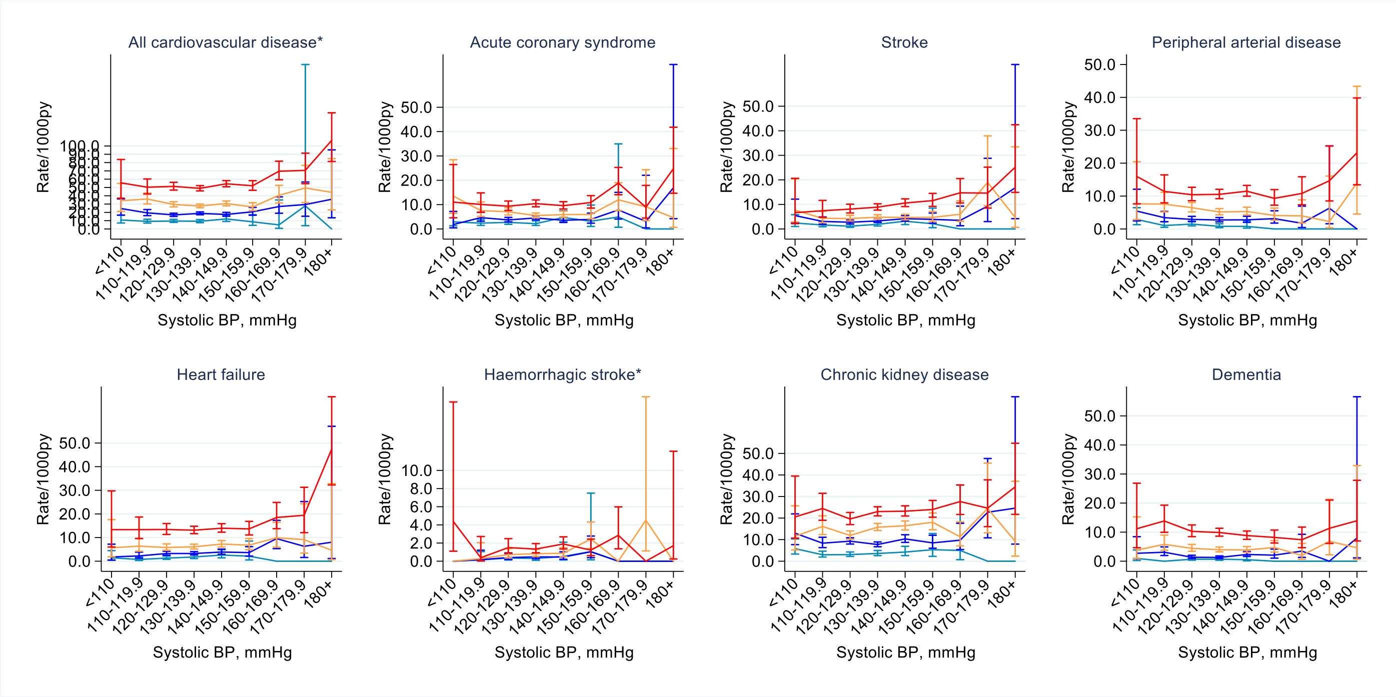

B. No diabetes at cohort entry

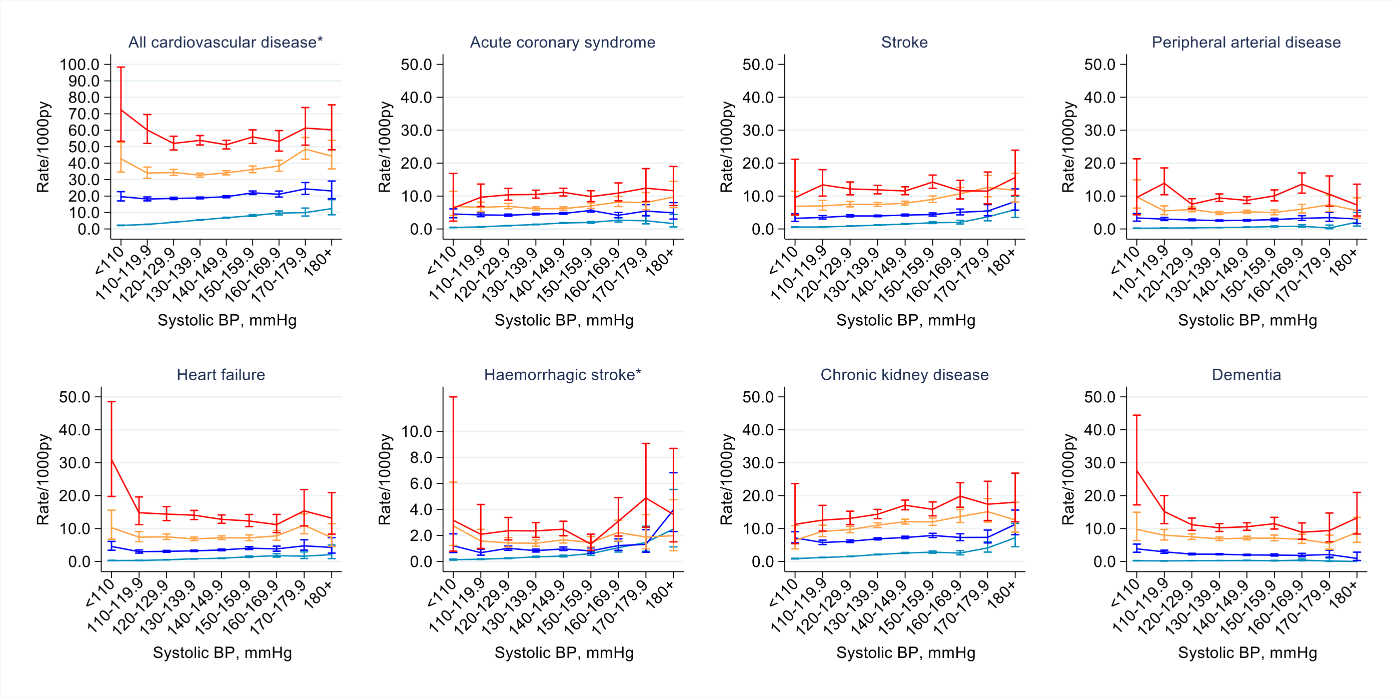

Appendix Figure 10. Among patients with (A) <u>diabetes at cohort entry</u> and (B) <u>no diabetes at cohort entry</u>, the rate (with 95% confidence interval) of cardiovascular disease, acute coronary syndrome, stroke, peripheral arterial disease, heart failure, haemorrhagic stroke, chronic kidney disease and dementia stratified by systolic blood pressure and predicted ten year cardiovascular disease risk (QRISK2) at cohort entry.

BP: blood pressure

*Note different scales on axes for all cardiovascular disease and haemorrhagic stroke. All cardiovascular disease includes all coronary heart disease, cerebrovascular disease, peripheral arterial disease and heart failure.

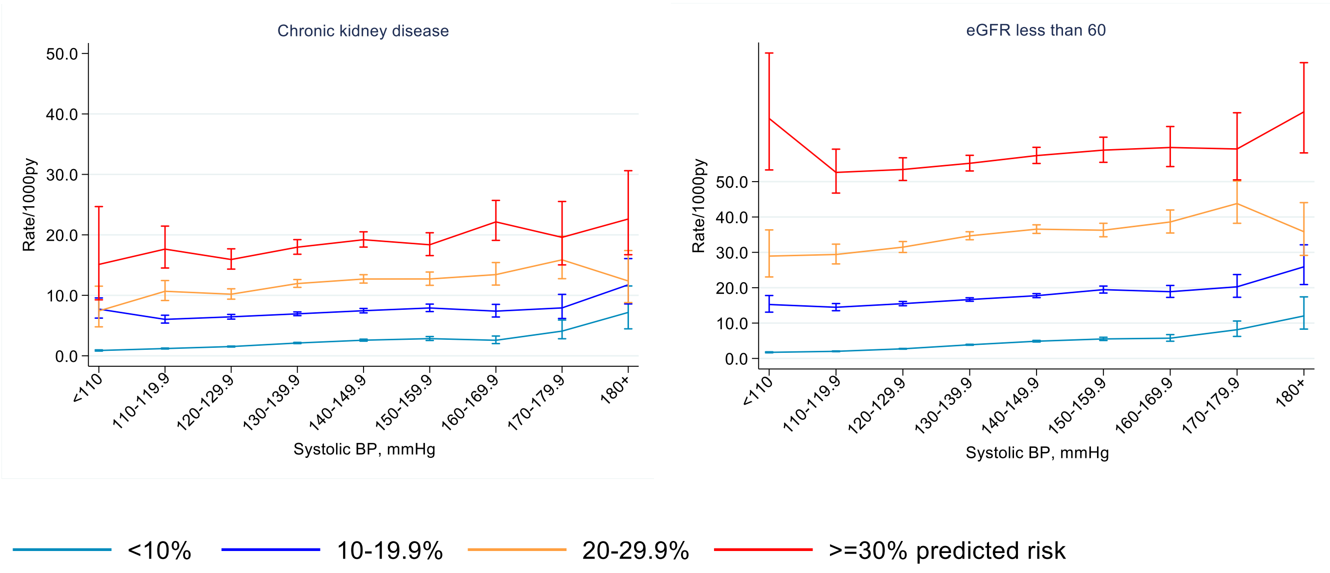

Appendix Figure 11. Rates of chronic kidney disease and eGFR<60ml/min/1.73 m2 during follow-up, stratified by systolic blood pressure and predicted ten year cardiovascular disease risk (QRISK2) at cohort entry.

BP: blood pressure

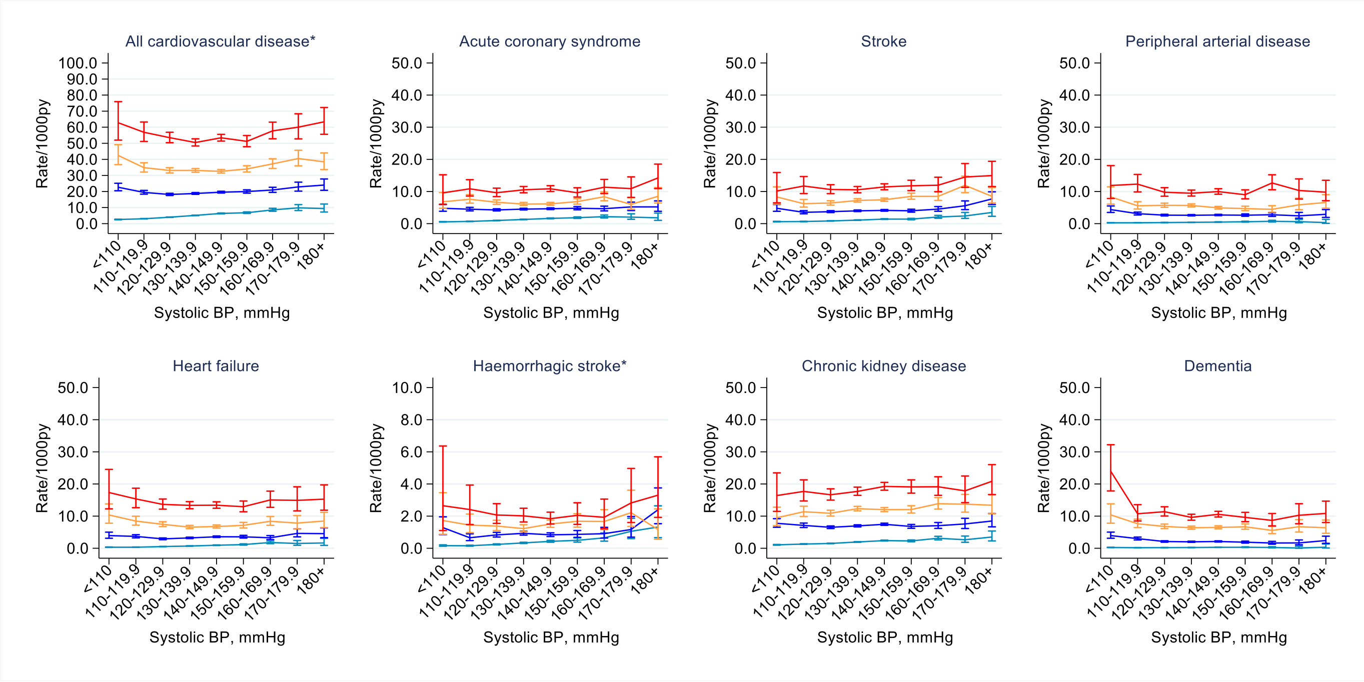

Appendix Figure 12. The rate (with 95% confidence interval) of cardiovascular disease, acute coronary syndrome, stroke, peripheral arterial disease, heart failure, haemorrhagic stroke, chronic kidney disease and dementia stratified by <u>most recent systolic blood pressure</u> and predicted ten year cardiovascular disease risk (QRISK2) at cohort entry.

BP: blood pressure

* All cardiovascular disease includes all coronary heart disease, cerebrovascular disease, peripheral arterial disease and heart failure.

Diastolic blood pressure

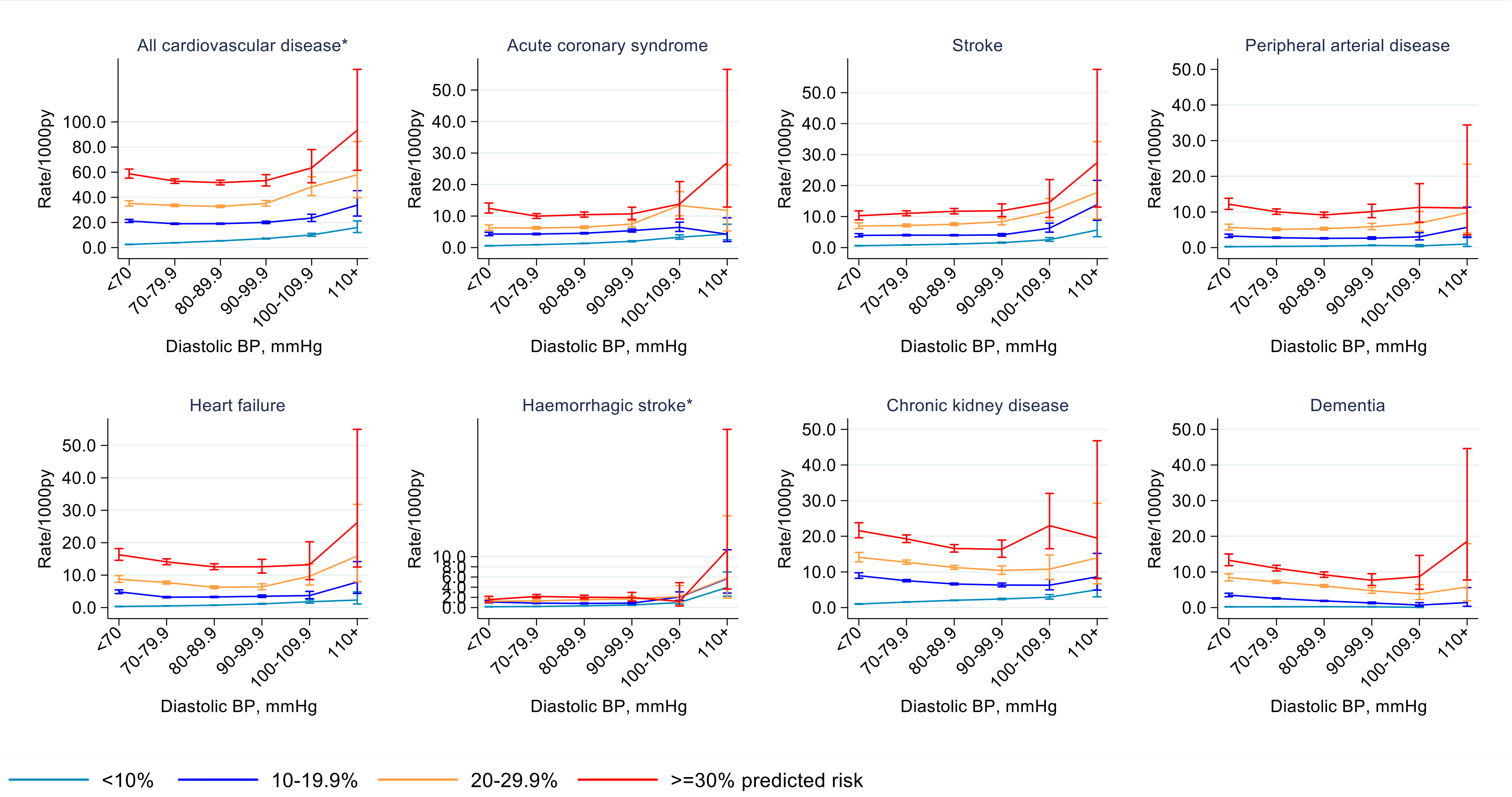

Figure 13. Rate (with 95% confidence interval) of cardiovascular disease, acute coronary syndrome, stroke, peripheral arterial disease, heart failure, haemorrhagic stroke, chronic kidney disease and dementia stratified by diastolic blood pressure and predicted ten year cardiovascular disease risk (QRISK2) at cohort entry.

BP: blood pressure

*Note different scales on axes for all cardiovascular disease and haemorrhagic stroke. All cardiovascular disease includes all coronary heart disease, cerebrovascular disease, peripheral arterial disease and heart failure.

Additional analyses – Methods, Results and Discussion

Calculation of estimated number needed to treat (NNT) to prevent one cardiovascular disease event

NNT Methods

Based on a recent meta-analysis of 74 trials, those with systolic blood pressures 160mmHg and above were assumed to gain a 22% treatment benefit, while those with blood pressures 140mmHg-159.9mmHg were assumed to have a 12% benefit. Patients with blood pressures below 140mmHg were assumed not to benefit from blood pressure lowering.[1] Calculation of NNTs was performed using direct standardization by treatment status, assuming those treated at cohort entry had already received treatment benefit, while those untreated at cohort entry were yet to benefit from treatment.[2] NNTs were presented for all cardiovascular disease outcomes and for each level of blood pressure (≥140mmHg) and predicted risk.

NNT Results

Estimated numbers needed to treat (NNT) to prevent an event

Restricting to patients with systolic blood pressures of 140mmHg and above, the NNTs for five years to prevent one cardiovascular disease outcome are shown in Figure 3. The NNT falls with increasing blood pressure, but much greater differences are observed between categories of predicted risk. For example, among patients with systolic blood pressure 160-169.9mmHg and predicted risk <10% (who would be treated under all global guidelines), we would need to treat an estimated 432 patients for five years to prevent one cardiovascular disease event. For patients with blood pressure 140-149.9mmHg but ≥30% predicted risk, we would need to treat an estimated 160 patients for five years to prevent one cardiovascular disease event.

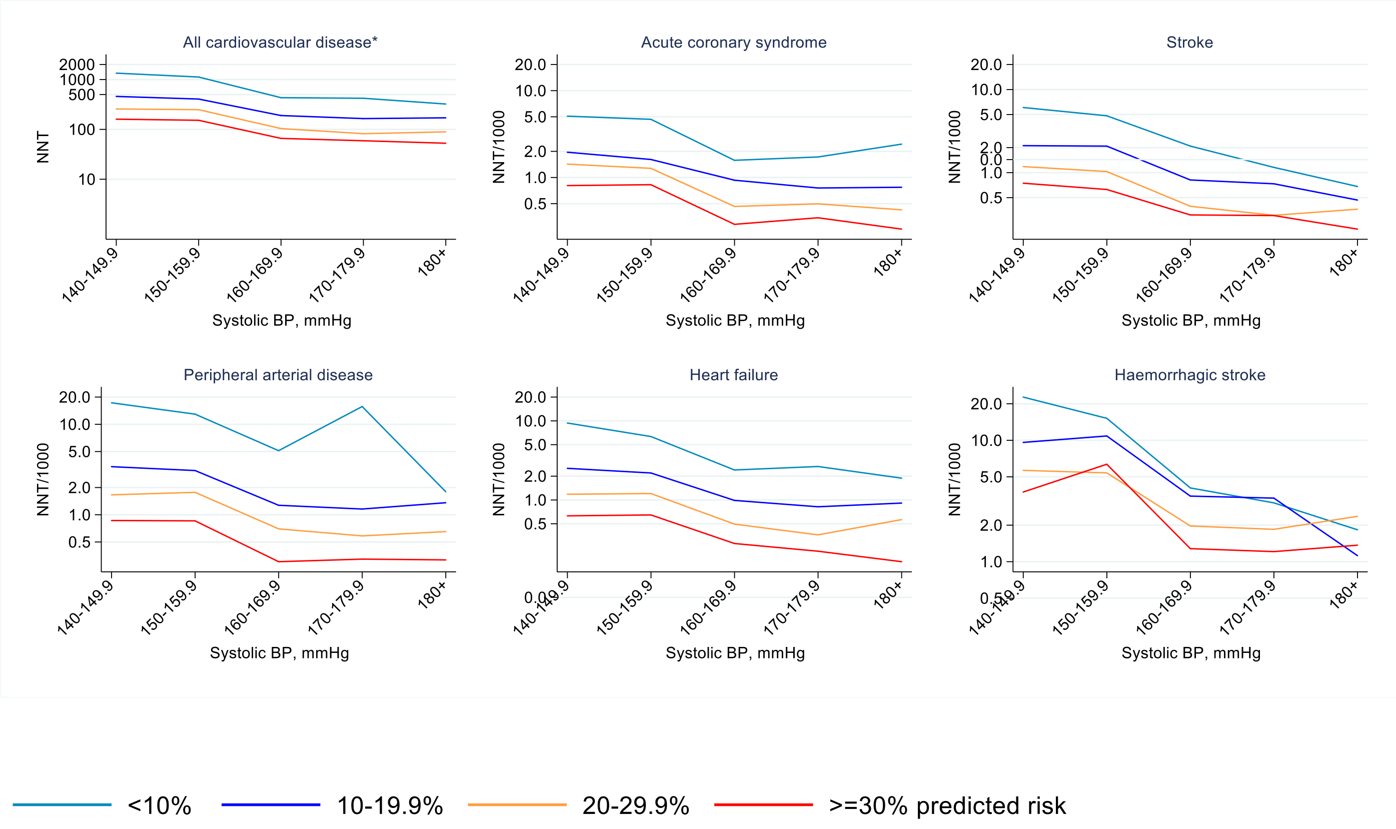

Figure 3. Number needed to treat for five years to prevent one cardiovascular disease, acute coronary syndrome, stroke, peripheral arterial disease, heart failure, haemorrhagic stroke, stratified by systolic blood pressure and predicted ten year cardiovascular disease risk (QRISK2) at cohort entry. All NNTs are plotted on the log scale. Based on effectiveness estimates from Brunstrom (blood pressure lowering treatment reduces cardiovascular disease by 22% if ≥160mmHg, but 12% if <160mmHg)[1]
BP: blood pressure
*Note the different scale on axis for all cardiovascular disease.
All cardiovascular disease includes all coronary heart disease, cerebrovascular disease, peripheral arterial disease and heart failure.

NNT Discussion

Three strong assumptions are required in our calculation of the number needed to treat, which were based on the rates among treated and untreated patients. First, that those on treatment all received full benefit from treatment, as estimated in meta-analysis.[1] This is likely to be an overestimation due to suboptimal adherence.[3,4] This would have made the outcome rates higher in the treated group than in a scenario of perfect adherence, and would have led to smaller differences between treated and untreated groups. Second, that those who were untreated did not receive any benefit from treatment. Our data showed that a large proportion of those untreated at baseline did subsequently receive treatment, which means that we may have overestimated the benefit among these patients. Third, that the effect of blood pressure lowering is the same across different cardiovascular disease outcomes. The effectiveness estimates used in the present study were based on those for major cardiovascular disease events[1] and it is unclear whether this would translate to all forms of acute and chronic cardiovascular disease.

References

1. Brunstrom M, Carlberg B. Association of Blood Pressure Lowering With Mortality and Cardiovascular Disease Across Blood Pressure Levels: A Systematic Review and Meta-analysis. JAMA internal medicine. 2018 Jan 1;178(1):28-36
2. Ettehad D, Emdin C, A. K, Anderson S, Callender T, Emberson J, et al. Blood pressure lowering for prevention of cardiovascular disease and death: a systematic review and meta-analysis. Lancet. 2015;http://dx.doi.org/10.1016/S0140-6736(15)01225-8
3. Dorans KS, Mills KT, Liu Y, He J. Trends in Prevalence and Control of Hypertension According to the 2017 American College of Cardiology/American Heart Association (ACC/AHA) Guideline. Journal of the American Heart Association. 2018 Jun 1;7(11)
4. Jung O, Gechter JL, Wunder C, Paulke A, Bartel C, Geiger H, et al. Resistant hypertension? Assessment of adherence by toxicological urine analysis. Journal of hypertension. 2013 Apr;31(4):766-74

The RECORD statement – checklist of items, extended from the STROBE statement, that should be reported in observational studies using routinely collected health data.

	Item No.	STROBE items	Location in manuscript where items are reported	RECORD items	Location in manuscript where items are reported
**Title and abstract**					
	1	(a) Indicate the study's design with a commonly used term in the title or the abstract (b) Provide in the abstract an informative and balanced summary of what was done and what was found	(a) Title<br><br>(b) Abstract	RECORD 1.1: The type of data used should be specified in the title or abstract. When possible, the name of the databases used should be included.<br><br>RECORD 1.2: If applicable, the geographic region and timeframe within which the study took place should be reported in the title or abstract.<br><br>RECORD 1.3: If linkage between databases was conducted for the study, this should be clearly stated in the title or abstract.	Abstract<br><br>Abstract<br><br>Abstract
**Introduction**					
Background rationale	2	Explain the scientific background and rationale for the investigation being reported			Introduction
Objectives	3	State specific objectives, including any prespecified hypotheses			Introduction
**Methods**					
Study Design	4	Present key elements of study design early in the paper			Methods
Setting	5	Describe the setting, locations, and relevant dates, including periods of recruitment, exposure, follow-up, and data collection			Methods
Participants	6	*(a) Cohort study* - Give the eligibility criteria, and the sources	Methods	RECORD 6.1: The methods of study population selection (such as codes or	Methods and

		and methods of selection of participants. Describe methods of follow-up<br>*Case-control study* - Give the eligibility criteria, and the sources and methods of case ascertainment and control selection. Give the rationale for the choice of cases and controls<br>*Cross-sectional study* - Give the eligibility criteria, and the sources and methods of selection of participants<br><br>*(b) Cohort study* - For matched studies, give matching criteria and number of exposed and unexposed<br>*Case-control study* - For matched studies, give matching criteria and the number of controls per case		algorithms used to identify subjects) should be listed in detail. If this is not possible, an explanation should be provided.<br><br>RECORD 6.2: Any validation studies of the codes or algorithms used to select the population should be referenced. If validation was conducted for this study and not published elsewhere, detailed methods and results should be provided.<br><br>RECORD 6.3: If the study involved linkage of databases, consider use of a flow diagram or other graphical display to demonstrate the data linkage process, including the number of individuals with linked data at each stage.	Flowchart of cohort selection in appendices<br><br>n/a<br><br>n/a
Variables	7	Clearly define all outcomes, exposures, predictors, potential confounders, and effect modifiers. Give diagnostic criteria, if applicable.	Methods	RECORD 7.1: A complete list of codes and algorithms used to classify exposures, outcomes, confounders, and effect modifiers should be provided. If these cannot be reported, an explanation should be provided.	Available in an online repository, link provided in the text.
Data sources/ measurement	8	For each variable of interest, give sources of data and details of methods of assessment (measurement).<br>Describe comparability of assessment methods if there is more than one group	Methods and online repository		
Bias	9	Describe any efforts to address potential sources of bias	Sensitivity analysis, Methods		

Study size	10	Explain how the study size was arrived at			Methods
Quantitative variables	11	Explain how quantitative variables were handled in the analyses. If applicable, describe which groupings were chosen, and why			Methods
Statistical methods	12	(a) Describe all statistical methods, including those used to control for confounding<br>(b) Describe any methods used to examine subgroups and interactions<br>(c) Explain how missing data were addressed<br>(d) *Cohort study* - If applicable, explain how loss to follow-up was addressed<br>*Case-control study* - If applicable, explain how matching of cases and controls was addressed<br>*Cross-sectional study* - If applicable, describe analytical methods taking account of sampling strategy<br>(e) Describe any sensitivity analyses			Methods
Data access and cleaning methods		..		RECORD 12.1: Authors should describe the extent to which the investigators had access to the database population used to create the study population.<br><br>RECORD 12.2: Authors should provide information on the data cleaning methods used in the study.	Data access statement<br><br>n/a

Linkage		..		RECORD 12.3: State whether the study included person-level, institutional-level, or other data linkage across two or more databases. The methods of linkage and methods of linkage quality evaluation should be provided.	Linkage details are provided by CPRD. Linkage was not performed by the study authors.
**Results**					
Participants	13	(a) Report the numbers of individuals at each stage of the study (*e.g.*, numbers potentially eligible, examined for eligibility, confirmed eligible, included in the study, completing follow-up, and analysed)<br>(b) Give reasons for non-participation at each stage.<br>(c) Consider use of a flow diagram		RECORD 13.1: Describe in detail the selection of the persons included in the study (*i.e.,* study population selection) including filtering based on data quality, data availability and linkage. The selection of included persons can be described in the text and/or by means of the study flow diagram.	Flowchart, appendix page 1
Descriptive data	14	(a) Give characteristics of study participants (*e.g.*, demographic, clinical, social) and information on exposures and potential confounders<br>(b) Indicate the number of participants with missing data for each variable of interest<br>(c) *Cohort study* - summarise follow-up time (*e.g.*, average and total amount)			Results and Table 1
Outcome data	15	*Cohort study* - Report numbers of outcome events or summary measures over time<br>*Case-control study* - Report numbers in each exposure category, or summary measures of exposure			Results

		Cross-sectional study - Report numbers of outcome events or summary measures			
Main results	16	(a) Give unadjusted estimates and, if applicable, confounder-adjusted estimates and their precision (e.g., 95% confidence interval). Make clear which confounders were adjusted for and why they were included<br>(b) Report category boundaries when continuous variables were categorized<br>(c) If relevant, consider translating estimates of relative risk into absolute risk for a meaningful time period			Results
Other analyses	17	Report other analyses done—e.g., analyses of subgroups and interactions, and sensitivity analyses			Results
**Discussion**					
Key results	18	Summarise key results with reference to study objectives			Discussion
Limitations	19	Discuss limitations of the study, taking into account sources of potential bias or imprecision. Discuss both direction and magnitude of any potential bias		RECORD 19.1: Discuss the implications of using data that were not created or collected to answer the specific research question(s). Include discussion of misclassification bias, unmeasured confounding, missing data, and changing eligibility over time, as they pertain to the study being reported.	Discussion
Interpretation	20	Give a cautious overall interpretation of results considering objectives, limitations, multiplicity			Discussion

		of analyses, results from similar studies, and other relevant evidence			
Generalisability	21	Discuss the generalisability (external validity) of the study results			Discussion
**Other Information**					
Funding	22	Give the source of funding and the role of the funders for the present study and, if applicable, for the original study on which the present article is based			Role of funding source
Accessibility of protocol, raw data, and programming code		..		RECORD 22.1: Authors should provide information on how to access any supplemental information such as the study protocol, raw data, or programming code.	Data access statement

*Reference: Benchimol EI, Smeeth L, Guttmann A, Harron K, Moher D, Petersen I, Sørensen HT, von Elm E, Langan SM, the RECORD Working Committee. The REporting of studies Conducted using Observational Routinely-collected health Data (RECORD) Statement. *PLoS Medicine* 2015.

*Checklist is protected under Creative Commons Attribution (CC BY) license.
